# Machine learning and SHAP value interpretation for predicting comorbidity of cardiovascular disease and cancer with dietary antioxidants

**DOI:** 10.1016/j.redox.2024.103470

**Published:** 2024-12-16

**Authors:** Xiangjun Qi, Shujing Wang, Caishan Fang, Jie Jia, Lizhu Lin, Tianhui Yuan

**Affiliations:** a The First Clinical Medical College, Guangzhou University of Chinese Medicine, Guangzhou, 510000, China; b Hospital of Chengdu University of Traditional Chinese Medicine, Chengdu University of Traditional Chinese Medicine, Chengdu, 610031, China; c Yong Loo Lin School of Medicine, National University of Singapore, 117597, Singapore; d The First Affiliated Hospital of Guangzhou University of Chinese Medicine, Guangzhou, 510405, China; e Guangdong Clinical Research Academy of Chinese Medicine, Guangzhou, 510405, China; f Kolling Institute of Medical Research, University of Sydney, Sydney, NSW, 2065, Australia

**Keywords:** Machine learning, SHAP, Cardiovascular disease, Cancer, Dietary antioxidants

## Abstract

**Objective:**

To develop and validate a machine learning model incorporating dietary antioxidants to predict cardiovascular disease (CVD)-cancer comorbidity and to elucidate the role of antioxidants in disease prediction.

**Methods:**

Data were sourced from the National Health and Nutrition Examination Survey. Antioxidants, including vitamins, minerals, and polyphenols, were selected as key features. Additionally, demographic, lifestyle, and health condition features were incorporated to improve model accuracy. Feature preprocessing included removing collinear features, addressing class imbalance, and normalizing data. Models constructed within the mlr3 framework included recursive partitioning and regression trees, random forest, kernel k-nearest neighbors, naïve bayes, and light gradient boosting machine (LightGBM). Benchmarking provided a systematic approach to evaluating and comparing model performance. SHapley Additive exPlanation (SHAP) values were calculated to determine the prediction role of each feature in the model with the highest predictive performance.

**Results:**

This analysis included 10,064 participants, with 353 identified as having comorbid CVD and cancer. After excluding collinear features, the machine learning model retained 29 dietary antioxidant features and 9 baseline features. LightGBM achieved the highest predictive accuracy at 87.9 %, a classification error rate of 12.1 %, and the top area under the receiver operating characteristic curve (0.951) and the precision‐recall curve (0.930). LightGBM also demonstrated balanced sensitivity and specificity, both close to 88 %. SHAP analysis indicated that naringenin, magnesium, theaflavin, kaempferol, hesperetin, selenium, malvidin, and vitamin C were the most influential contributors.

**Conclusion:**

LightGBM exhibited the best performance for predicting CVD-cancer comorbidity. SHAP values highlighted the importance of antioxidants, with naringenin and magnesium emerging as primary factors in this model.

## 1 Introduction

The phenomenon of cardiovascular disease (CVD) and cancer comorbidity has received increasing attention, as these two disease types often share common pathological mechanisms, including oxidative stress, inflammatory responses, and immune dysregulation [1,2]. Oxidative stress not only causes cellular damage but also promotes atherosclerosis and cancer cell proliferation, invasion, and metastasis through chronic inflammation, thereby increasing the comorbidity risk for CVD and cancer patients [3]. CVD and cancer risk are positively correlated, and conversely, cancer and CVD risk also show a positive association. Using IBMMarketScan claims data from over 130 million individuals, 27 million cancer-free subjects with at least 36 months of follow-up were identified. Among these 27,195,088 individuals, those with CVD had a 13 % higher likelihood of developing cancer compared to those without CVD [4]. Significant advancements in cancer treatment have markedly improved patient prognosis. However, cancer survivors face increased risks of CVD and cardiovascular mortality, a conclusion supported by studies involving 4,519,243 adults in Canada [5] and data from the Surveillance, Epidemiology, and End Results program in the United States [6,7]. These findings underscore the importance of research into the prevention and management of CVD-cancer comorbidity [8].

Dietary antioxidants (such as flavonoids, vitamins, and polyphenolic compounds) mitigate oxidative stress by neutralizing free radicals, potentially providing significant protective effects against these diseases [9]. In the context of CVD-cancer comorbidity, the preventive role of dietary antioxidants has received considerable attention in recent years. While studies have shown that dietary antioxidants are associated with a reduced risk of individual diseases (CVD or cancer) [10], their specific protective role in CVD-cancer comorbidity risk remains unclear. Therefore, further investigation into the protective effects of dietary antioxidants in this complex pathology is of great importance.

Our study aimed to utilize the National Health and Nutrition Examination Survey (NHANES) to identify potential associations between dietary antioxidant intake and CVD-cancer comorbidity through machine learning (ML) methods. Unlike traditional statistical methods, ML techniques can handle large, complex datasets and identify implicit relationships among various health-related features, thereby predicting disease risk more accurately [11]. Our study employed benchmarking to compare model performance and SHapley Additive exPlanation (SHAP) values to enhance the interpretability of the model, revealing the specific contributions of each dietary antioxidant.

## 2 Participants and methods

### 2.1 Participants

**Fig. 1:**
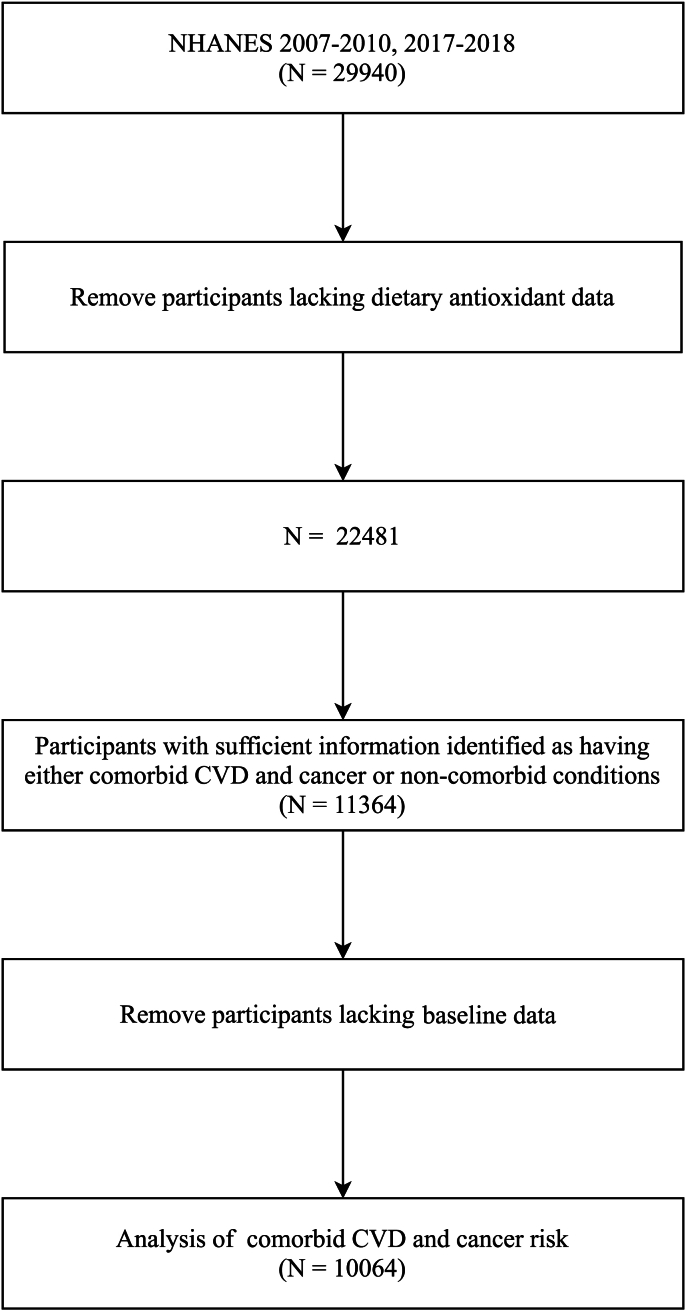
Participants screening flow chart.

The NHANES, conducted by the National Center for Health Statistics, gathers demographic, socioeconomic, dietary, and health-related data for health assessment. Participants in NHANES 2007–2010 and NHANES 2017–2018 were considered candidates for this study. Individuals with complete information about dietary antioxidant intake and diagnosis of CVD and cancer were included in the study. Participants with missing baseline features data were excluded. Refer to Fig. 1 for detailed participant screening procedures.

### 2.2 Dietary antioxidant intake

The intake data for 44 dietary antioxidants, including vitamins, minerals, and polyphenols, were obtained from NHANES. Participants undergo two 24-h dietary recall interviews at a Mobile Examination Center, with a 3–10 day interval between interviews. The average daily intake of dietary antioxidants was calculated.

### 2.3 Diagnosis of CVD and cancer

CVD diagnosis was confirmed if participants reported being informed by a physician or other healthcare professional that they had congestive heart failure, coronary heart disease, angina, myocardial infarction, or stroke. Similarly, a cancer diagnosis was confirmed if participants reported being informed by a physician or other healthcare professional that they had cancer, specified a particular type of cancer in the medical conditions questionnaire, or reported the use of anti-cancer medications.

### 2.4 Collection of baseline features

Baseline characteristics were collected based on three aspects including demographic, lifestyle, and health condition. These included age, gender (male or female), race/ethnicity (Mexican American, other Hispanic, non-Hispanic white, non-Hispanic black, and other categories), family poverty-to-income ratio (classified as 0–1, 1–3, or >3), smoking status (never, former, or current), engagement in moderate to vigorous physical activity (Yes or No), as well as the presence of hyperlipidemia, hypertension, and diabetes. Data on age, gender, race/ethnicity, and family poverty-to-income ratio were retrieved from the Demographic Data module in NHANES, while information on smoking and physical activity was sourced from the Questionnaire Data module. Individuals who reported smoking fewer than 100 cigarettes over their lifetime were categorized as never smokers, while others were classified as former or current smokers based on their response to the question “Do you now smoke cigarettes?“. The diagnoses of hyperlipidemia, hypertension, and diabetes were established using both laboratory measurements and self-reported data from the Questionnaire Data module. Specifically, hyperlipidemia was defined by high-density lipoproteincholesterol levels below 1.0 mmol/L in men, below 1.3 mmol/L in women, or triglycerides at or above 1.8 mmol/L for all participants. Hypertension was identified as having a systolic blood pressure ≥130 mmHg and/or diastolic blood pressure ≥80 mmHg on at least three occasions, or if participants answered “yes” to questions about taking prescribed medication for high blood pressure or being previously diagnosed with hypertension. Diabetes was determined through a positive response to the question “Doctor told you have diabetes?” or meeting one or more of the following criteria: glycohemoglobin ≥6.5 %, fasting glucose ≥7 mmol/L, 2-h blood glucose ≥11.1 mmol/L during an oral glucose tolerance test, random serum glucose ≥11.1 mmol/L, or use of anti-diabetic medication.

### 2.5 Pre-processing of machine learning features

The dataset for this study initially consisted of 55 features, comprising 46 continuous variables and 9 categorical ones. To reduce multicollinearity among dietary antioxidant features, correlation coefficients were calculated, and features with coefficients exceeding 0.9 were removed. To mitigate the issue of class imbalance between comorbidity and non-comorbidity groups, the Synthetic Minority Over-sampling Technique was applied. This technique generates synthetic samples for the minority class by interpolating new data points along the line segments that connect each minority class sample to its K-nearest neighbors. Finally, we standardized all features using the Standard Scaler to prevent features with larger numerical values from disproportionately influencing the model's performance during training.

### 2.6 Statistical analysis

The characteristics of participants with and without comorbidity disease were described using survey-weighted statistical models. Continuous variables were presented as mean ± standard error, while categorical variables were expressed as frequencies and percentages. Characteristics were compared using the weighted χ^2^ test for categorical variables, ANOVA for normally distributed continuous variables, and the Kruskal-Wallis H test for skewed distributions.

Discrimination models including recursive partitioning and regression trees (RPART), random forest (RF), Kernel k-nearest neighbors (K–KNN), naïve bayes (NB), and light gradient boosting machine (LightGBM) were constructed under the mlr3 framework. The RPART model can capture nonlinear relationships and feature interactions, making it suitable for complex variable relationships in data [12]. RF, an ensemble learning method, significantly reduces the risk of overfitting by constructing multiple decision trees and averaging their predictions. It also handles complex interactions between features more effectively [13]. The K–KNN model classifies samples based on their similarities, making it suitable for data with uneven sample distributions [14]. NB is computationally efficient when handling large-scale data [15]. LightGBM, an efficient implementation of gradient boosting, offers significant computational advantages when processing large-scale data. By utilizing gradient-based one-side sampling and exclusive feature bundling, it can train models rapidly while maintaining high prediction accuracy [16]. These models also have been successfully applied in the analysis of NHANES data in previous studies [[17], [18], [19]], demonstrating their applicability.

Benchmarking serves as a crucial methodology for systematically evaluating and comparing the performance of ML models. This process involves assessing multiple models on standardized datasets and using consistent evaluation metrics to ensure a fair comparison. Metrics including classification error rate, accuracy, F-beta, area under the receiver operator characteristic (ROC) curve, sensitivity, specificity and area under the recision-recall (PR) curve were selected based on the nature of the classification task. A higher area under the ROC curve is an important indicator for selecting the best model, while other metrics serve as supplementary tools for assessing model performance. To reduce the assessment bias of the ML models, data resampling was performed using 10-fold cross-validation. ANOVA and the Kruskal-Wallis H test are used to examine the differences in metrics across different models.

We utilized SHAP values to assess the overall feature importance in the ML model with the best predictive performance. SHAP, a recent advancement in making tree-based models more interpretable, employs a game-theoretic method that aggregates the local contributions of individual features to explain the model's behavior on a global scale. This approach is considered superior to other global approximation methods. The algorithm not only provides a measure of feature importance across the model but also offers insights into the role of each feature in specific predictions.

Data analysis was performed with the statistical software package R (v4.4.1). The R packages *survey*, *DMwR*, *ggcor*, *mlr3*, *mlr3benchmark*, *mlr3extralearner*, *kernelshap*, and *shapviz* were utilized for statistical analysis. Statistical tests were two-sided and a *p*-value < 0.05 was considered statistically significant.

## 3 Results

### 3.1 Characteristics of the features

A total of 10,064 participants were included in this analysis, and 353 of them were identified as having comorbidities of CVD and cancer. Compared to participants without comorbidities, those with comorbid conditions had significantly lower intakes of magnesium (Mg) (260.893 [6.131] vs. 302.756 [2.976]), Zinc (10.489 [0.328] vs. 11.765 [0.133]), selenium (Se) (97.070 [3.122] vs. 114.166 [0.835]) and isorhamnetin (0.730 [0.067] vs 0.888 [0.023]). There were significant differences between the two groups in terms of age, body mass index, racial composition, degree of education, diabetes condition, hyperlipidemia condition, hypertension condition, physical activity, and smoking status (Table 1).

**Table 1:** Metrics of the 5 machine learning models in predicting CVD and cancer comorbidities.

Machine learner	Classification error rate	Accuracy	F-beta	Area under the ROC curve	Sensitivity	Specificity	Area under the PR curve
RPART	0.201	0.799	0.767	0.831	0.772	0.822	0.767
RF	0.124	0.876	0.859	0.949	0.878	0.874	0.925
K–KNN	0.191	0.809	0.798	0.883	0.883	0.752	0.803
NB	0.350	0.650	0.682	0.802	0.876	0.479	0.751
LightGBM	0.121	0.879	0.862	0.951	0.880	0.878	0.930
*P*-value	<0.001^a^	<0.001^a^	<0.001^a^	<0.001^b^	<0.001^a^	<0.001^a^	<0.001^a^

RPART: recursive partitioning and regression trees; RF: random forest, K–KNN: Kernel k-nearest neighbors, NB: naïve bayes; LightGBM: light gradient boosting machine.

a ANOVA test.

b Kruskal-Wallis.

### 3.2 Development and validation of the comorbidity disease prediction model

**Fig. 2:**
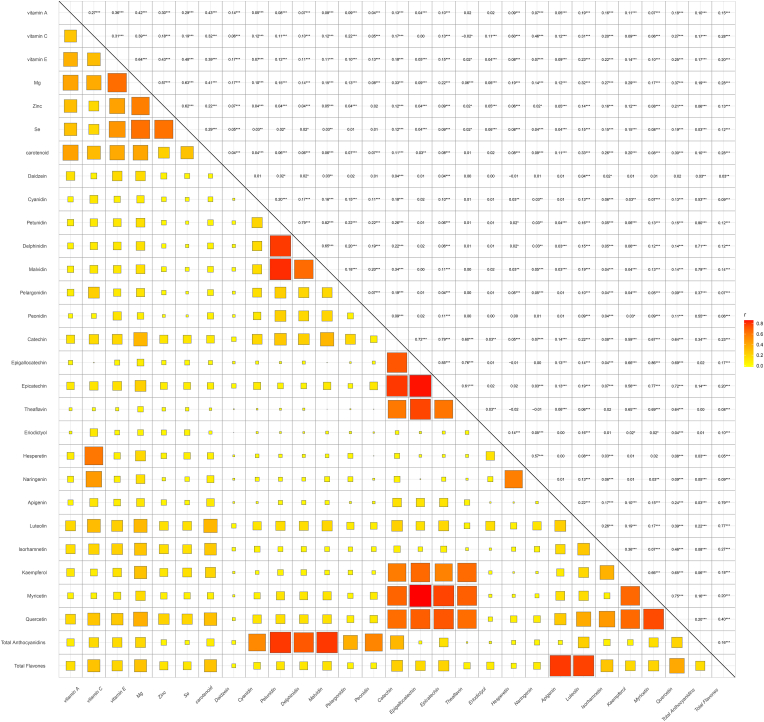
Correlation among remaining dietary antioxidant features after removing highly correlated ones.

Before constructing the ML model, we visualized the feature distributions. The distribution of categorical features is shown in Supplementary Fig. 1, and the distribution of continuous features is shown in Supplementary Fig. 2. The correlation coefficients between dietary antioxidant features are displayed in Supplementary Fig. 3. From Supplementary Fig. 3, it is evident that some dietary antioxidant features exhibit high correlations, such as genistein, epicatechin-3-gallate, epigallocatechin, theaflavin-3-3-digallate, theaflavin-3q-gallate, and theaflavin-3-gallate. Fig. 2 illustrates the dietary antioxidant features included in the ML model after addressing collinearity. Finally, the ML model included 29 dietary antioxidant features and 9 baseline features.

**Fig. 3:**
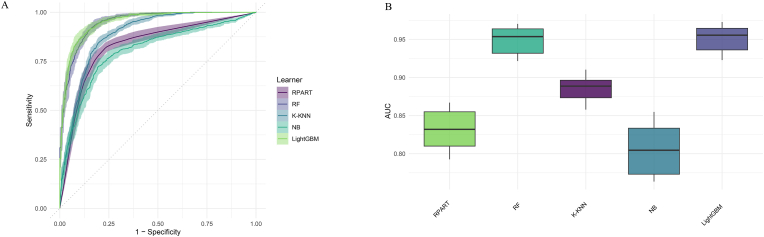
Receiver operating characteristic curves for the 5 machine learning models in predicting cardiovascular disease and cancer comorbidity. (A). Receiver operating characteristic curves. (B). Areas under the receiver operating characteristic curves.

**Fig. 4:**
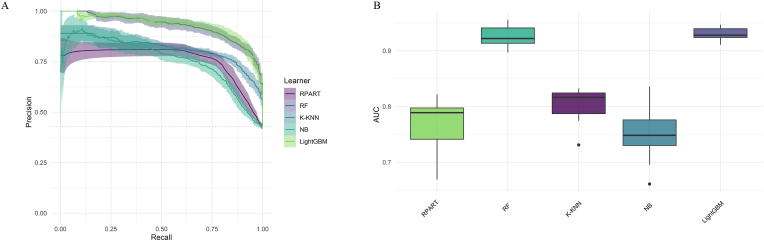
Precision-recall curves for the 5 machine learning models in predicting cardiovascular disease and cancer comorbidity. (A) Precision-recall curves. (B) Areas under the precision-recall curves.

Table 1 presents a detailed evaluation of five ML models—RPART, RF, K–KNN, NB, and LightGBM—assessed on several performance metrics including sensitivity (Supplementary Fig. 4), specificity (Supplementary Fig. 5), F-beta score (Supplementary Fig. 6), accuracy (Supplementary Fig. 7), classification error rate (Supplementary Fig. 8), area under the ROC curve (Fig. 3), and area under the PR curve (Fig. 4). Among the models, LightGBM stands out with the highest accuracy at 87.9 %, indicating its ability to correctly classify most instances. It also shows the lowest classification error rate of 12.1 %, making it the most effective in reducing misclassifications. LightGBM further achieves the highest values in both area under the ROC curve (0.951) and the PR curve (0.930), highlighting its superior ability to distinguish between classes and perform well under varying precision-recall conditions. The Random Forest model closely follows with an accuracy of 87.6 % and a similarly strong performance in the ROC (0.949) and PR curves (0.925). Both LightGBM and RF exhibit balanced sensitivity and specificity, with values close to 88 % for LightGBM, making them highly reliable for the task. In contrast, Naïve Bayes shows the weakest performance, with the lowest accuracy of 65.0 % and a classification error rate of 35.0 %. Its specificity is notably low (47.9 %), indicating that it struggles to correctly identify negative cases, despite its reasonable sensitivity (87.6 %). RPART and K–KNN offer moderate performance, with accuracies of 79.9 % and 80.9 %, respectively. However, K–KNN demonstrates higher sensitivity (88.3 %) compared to RPART (77.2 %), while RPART shows better specificity at 82.2 %. Furthermore, there are significant statistical differences in the metrics of the different models.

### 3.3 Importance of dietary antioxidant features interpreted by SHAP value

**Fig. 5:**
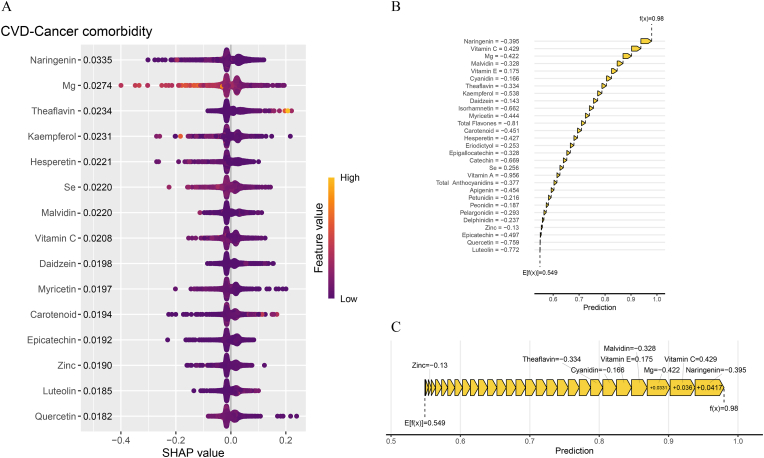
SHAP values of dietary antioxidants for LightGBM model. (A). SHAP summary plot. (B) SHAP waterfall plot. (C). SHAP force plot.

The SHAP plot (Fig. 5A and Supplementary Fig. 9) shows the importance of each feature (Top 15 sorted by importance) in the machine model for predicting comorbidity disease. SHAP values indicate that naringenin (0.0335), Mg (0.0274), theaflavin (0.0234), kaempferol (0.0231), hesperetin (0.0221), Se (0.0220), malvidin (0.0220) and vitamin C (0.0208) were the major negative contributors. To better illustrate the contribution of dietary antioxidants in the model's predictions and the prediction process, we used the *shapviz* package to generate waterfall plots (Fig. 5B) and force plots (Fig. 5C). Fig. 5B shows the contribution ranking of antioxidants in predicting non-comorbidity events, as well as the cumulative prediction level, with the final prediction reaching 0.98. In Fig. 5C, all dietary antioxidants in orange represent features that contribute to a lower risk of comorbidity events. These visualizations provide users with detailed insights into how the model makes predictions, allowing them to make informed dietary modifications. Additionally, we plotted the SHAP values and the correlation scatter plot between dietary antioxidant features (Supplementary Fig. 10). From the scatter plot, it can be seen that naringenin, Mg, vitamin C, vitamin E, Se, apigenin, kaempferol, myricetin, and quercetin exhibit a positive correlation with SHAP values.

## 4 Discussion

We used interpretable ML methods to study the relationship between dietary antioxidant intake and CVD-cancer comorbidity in the U.S. NHANES dataset from 2007 to 2010 and 2017–2018. Among the 5 ML models considered, the LightGBM model performed the best. The LightGBM model was tested with an average AUC of 0.951, indicating excellent efficiency and stability for classification. Using the SHAP game-theoretic approach illustrated the importance of each selected feature in the model, with naringenin, Mg, theaflavin, kaempferol, hesperetin, Se, malvidin and vitamin C being the major contributors.

To the best of our knowledge, this is the first study to develop and validate a CVD-cancer comorbidity prediction model incorporating antioxidant factors alongside baseline characteristics. Although our primary focus was to analyze the contribution of dietary antioxidants, the model also integrates easily accessible demographic characteristics, lifestyle factors, and individual health conditions, which enhance model simulation. Additionally, benchmarking was employed to compare the performance of various models.

ML models have increasingly been used to explore dietary factors associated with cardiovascular disease and cancer. Ravi V. Shah and colleagues, for example, used penalized ML models to integrate dietary and metabolic factors (e.g., fish and long-chain unsaturated triacylglycerols) and developed a dietary-metabolic model for predicting diabetes-CVD risk [20]. Similarly, Agustin Martin-Morales et al. applied ML models to predict cardiovascular mortality by comparing logistic regression, support vector machine, RF, XGBoost, and LightGBM, ultimately selecting RF as the optimal model. SHAP analysis highlighted age, systolic blood pressure, and various health indicators as essential variables, with dietary components like fiber, calcium, and vitamin E contributing to improved model performance [21]. Guadalupe Gutiérrez-Esparza and colleagues employed Variable Importance Measures using RF, XGBoost, and Gradient Boosting Machine to assess factors such as anthropometric measurements, biochemical tests, dietary intake, and family health history for dyslipidemia, though the model achieved an accuracy of only 80 % [22]. In studies linking diet and cancer, dietary factors alone have yielded satisfactory predictive models. Hanif Abdul Rahman et al. consolidated data from 109,343 participants across Canada, India, Italy, Korea, Mexico, Sweden, and the USA, employing nine supervised and unsupervised ML models to predict colorectal cancer (CRC). An artificial neural network model achieved a misclassification rate of 1 % for CRC and 3 % for non-CRC cases [23]. Meanwhile, Noura Qarmiche used clustering to categorize dietary patterns as “dangerous” or “prudent.” While these studies included a broad range of dietary characteristics, another study exclusively examined dietary antioxidant properties [24]. Jiaqi Yang analyzed the association between dietary and supplemental antioxidants and lung cancer using a random forest model to evaluate antioxidant importance. Their findings indicated that α-carotene, Mg, vitamin C, vitamin E, Se, luteolin and zeaxanthin, and β-carotene exerted the most beneficial effects on lung cancer prevention [25]. These studies illustrate the application of ML in exploring associations between dietary factors and individual diseases, providing valuable insights for the CVD-cancer comorbidity field. They also applied multiple models but generally lacked benchmarking for comparative evaluation of model performance.

We selected RPART, RF, K–KNN, NB, and LightGBM to construct prediction models and evaluated the distinguishing features of each with benchmarking to determine the most suitable model for predicting CVD-cancer comorbidity. Compared to traditional statistical methods, such as logistic regression, machine learning approaches can provide deeper insights into this topic. They offer the following advantages: Machine learning algorithms can directly capture nonlinear and complex interactions without the need for multi-step statistical analyses. Algorithms like RF and LightGBM can automatically identify the most important predictive features, reducing the subjectivity involved in manual feature selection. In large-scale healthcare datasets, machine learning algorithms are more effective and help mitigate the risk of overfitting. Unlike statistical models with strict assumptions, machine learning algorithms impose fewer requirements on data distribution and can adapt to various types of healthcare data.

Our research indicates that the LightGBM model is optimal. LightGBM was designed as an efficient and robust gradient boosting model, particularly well-suited for handling large, complex datasets. It uses a leaf-wise growth strategy based on decision trees, combined with techniques such as Gradient-based One-Side Sampling GOSS and Exclusive Feature Bundling EFB, giving it significant training speed and memory efficiency on large-scale data. These features ensured LightGBM's accuracy and adaptability when processing data, enabling it to perform well even in environments with unstructured data, though light preprocessing was typically required to achieve optimal results [26]. The LightGBM model has been applied in various electronic health records (EHRs) for constructing disease prediction models. Siru Liu et al. [27] developed a machine learning model to predict new onset delirium, incorporating a total of 331,489 confusion assessment method assessments with 896 features from 34,035 patients' EHRs. Compared to logistic regression, random forest, support vector machine, and neural network algorithms, the LightGBM model demonstrated the best predictive performance (AUC 0.927). Similarly, in another study by Suparno Datta et al. [28], EHRs of 233,895 adult patients were used to build a machine learning model to predict hypertension, with LightGBM showing comparable predictive power to XGBoost and long short-term memory models. Zheyi Dong et al. developed a machine learning model to predict the 3-year risk of diabetic kidney disease [29], in which they included a smaller sample of only 816 patients' EHRs. Compared to models like extreme gradient boosting, adaptive boosting, artificial neural networks, decision trees, support vector machines, and logistic regression, the LightGBM model exhibited the best predictive ability (AUC 0.815). Notably, LightGBM can be further improved according to specific needs. Yan Wang et al. developed a modified LightGBM model, called HY_LightGBM, to predict blood glucose levels [30]. This model optimizes parameters using a Bayesian hyper-parameter optimization algorithm based on LightGBM, providing superior predictive performance in this context. These studies demonstrate that the LightGBM model is a high-quality machine learning model for both large and small sample EHRs and has the potential for algorithm improvements based on specific scenarios. These characteristics make it a valuable tool for providing deeper insights into healthcare services.

The reasons for the strong performance of the LightGBM model in our study may include the following: Firstly, LightGBM is well-suited for high-dimensional sparse data. In dietary antioxidant intake data, a zero intake is common, and LightGBM utilizes a histogram-based decision tree method that discretizes continuous features during data splitting, allowing the model to effectively handle sparse data. Secondly, among the features included in our study, there are both continuous and categorical variables. However, not all machine learning models support mixed-type features, whereas LightGBM does. Furthermore, LightGBM does not require one-hot encoding or label encoding for categorical features, which helps preserve the true distribution of these features in the model. Thirdly, LightGBM is well-suited for handling imbalanced data. Although we addressed the imbalance during data preprocessing, a model adept at handling imbalanced data will be more appropriate for this task.

To improve the interpretability and intuitiveness of ML methods, we applied SHAP values to the LightGBM model to enhance its interpretability and the impact assessment of key features. SHAP values are widely recognized in ML, especially in the medical field, such as for predicting cardiovascular disease, where they provide powerful interpretive capabilities. SHAP values can evaluate the effect of each feature on model outputs [17]. SHAP decision plots further helped us visualize the individual decision-making process within the LightGBM model. The results indicated that polyphenols (such as naringenin, theaflavin, kaempferol, hesperidin, and malvidin), minerals (such as Mg and Se), and vitamins (such as vitamin C) were major factors.

Existing studies support the positive role of antioxidants in mitigating CVD-cancer comorbidity. Oxidative stress is a component of cardiovascular disease and cancer, making targeting oxidative stress a promising disease prevention strategy. Although the mechanisms by which oxidative stress contributes to disease onset and progression are not yet fully understood, it is currently believed that oxidative stress leads to disease through two primary mechanisms. The first involves the production of reactive species during oxidative stress—particularly hydroxyl radical, peroxynitrite, and hypochlorous acid—which directly oxidize macromolecules, including membrane lipids, structural proteins, enzymes, and nucleic acids, leading to cellular dysfunction and death [31]. The second mechanism of oxidative stress is abnormal redox signaling. Redox signaling depends on the specific interactions between signaling proteins and hydrogen peroxide (H_2_O_2_), or other electrophilic agents that act as secondary messengers. Physiologically, H_2_O_2_ produced under stimulation can function as a secondary messenger [32]. In oxidative stress, non-physiological levels of H_2_O_2_ disrupt redox signaling [33]. The balance between oxidants and reductants, including glutathione, thioredoxin, and nicotinamide adenine dinucleotide phosphate, which serve as substrates for antioxidant enzymes, is essential for maintaining normal physiological functions [34]. These two mechanisms can exist independently or simultaneously during the onset and progression of disease.

In this study, the dietary antioxidant features mainly consist of three categories: vitamins, minerals, and polyphenols. These small molecules exert their antioxidant effects through different mechanisms. Vitamin C exerts its antioxidant effects by providing an electron to neutralize free radicals [35]. Vitamin E reduces peroxyl radicals and forms tocopheroxyl radicals, further regulating the bioactivity and signaling related to membrane lipids [36]. Minerals primarily function as cofactors for enzymes involved in oxidative stress; however, their roles are not limited to this. For example, selenium and zinc are cofactors for glutathione peroxidase [37], and Mg is a cofactor for glutathione peroxidase, superoxide dismutase, and catalase [38]. Dietary polyphenols are powerful antioxidants in vitro, capable of neutralizing free radicals by donating an electron or hydrogen atom to a wide range of reactive oxygen, nitrogen, and chlorine species, including O2^-^, hydroxyl radical, peroxyl radicals, hypochlorous acid, and peroxynitrous acid [39].

Naringenin and Mg are the two most important antioxidants in this study. Naringenin, a flavonoid compound [40], is widely found in yeasts, plants, and fungi [41]. Although no clinical trials currently validate its efficacy in preventing or treating cardiovascular disease, preclinical studies demonstrate its benefits, including inhibiting foam cell formation in vascular walls [42], reducing vascular smooth muscle cells migration and neointimal hyperplasia [43], suppressing reactive oxygen species generation [44], lowering angiotensin converting enzyme-1, and angiotensin II levels in myocardial tissue [45], and reducing ischemic and I/R-injured areas [46]. These effects are associated with antioxidative stress, anti-inflammatory, and anti-apoptotic mechanisms [47]. Similarly, naringenin exhibits broad-spectrum anticancer properties. Cellular and animal studies show that naringenin effectively blocks tumor cell cycles and inhibits tumor cell proliferation in multiple cancers, including cervical cancer [48], prostate cancer [49], breast cancer [50], CRC [51], and bladder cancer [52]. Moreover, naringenin is involved in mediating apoptosis and autophagy in tumor cells, as well as inhibiting tumor invasion and metastasis [53]. Mg ranks second in importance by SHAP value. As the most abundant divalent cation intracellularly, Mg is essential for maintaining cellular physiology and metabolism, acting as a cofactor for numerous enzymes, regulating ion channels, and supporting energy production [54]. In the heart, Mg plays a key role in neuronal excitability, cardiac conduction, and myocardial contraction by regulating ion transporters, including potassium and calcium channels [54,55]. Observational data show that low serum Mg levels or dietary intake are associated with an increased risk of atherosclerosis [56], coronary artery disease [57], arrhythmias [58], and heart failure [59]. A meta-analysis of epidemiologic studies showed that the relative risk (RR) of overall cancer for the highest level of dietary Mg intake was 0.801 [95 % confidence interval (CI): 0.664–0.966] compared with the lowest intake level [60]. In specific cancer types, such as lung cancer, a systematic review and meta-analysis indicated a significant association between Mg intake and reduced incidence (RR = 0.88, 95 % CI = 0.79–0.98) [61]. Similar findings were observed for colorectal cancer [62].

The current study is related to clinical practice in several aspects. First, our model achieved a satisfactory disease prediction capability, which suggests that in the future, dietary component surveys could be used to assess participants' disease risks, providing a non-invasive method for evaluation. Second, our study highlights the importance of dietary antioxidant features. As modifiable factors, the model results can be used to design healthy dietary intervention plans aimed at reducing potential disease risks. Finally, although many small molecules evaluated as antioxidants have shown therapeutic potential in preclinical studies, the clinical trial outcomes have been disappointing [31]. Our study may provides some new insights for the development of subsequent research.

Our study has several limitations. The diagnoses of CVD and cancer were partly based on self-reported data from the NHANES interview questionnaire, which could introduce information bias due to potential recall issues or cognitive limitations. Dietary differences across different populations and regions may influence the model's predictions. However, due to the lack of relevant data, we were unable to conduct further analysis on this aspect. Cross-sectional data is insufficient to reveal the causal relationship. Future longitudinal studies will help us better establish machine learning models, which is promising. This study utilized data from the U.S. NHANES dataset, a nationally representative sample. In model construction, in addition to incorporating antioxidant features, factors such as gender, race, income, lifestyle, and health status were also included. The inclusion of these factors enhances the generalizability of the results. However, the differences in dietary habits and health conditions across different countries limit the external validity of the findings. Therefore, future research should consider model training and validation in the context of different countries and dietary backgrounds. Additionally, the complexity and interpretive challenges of the models may limit their reproducibility and practical application in this context. SHAP values are designed to explain the contribution of individual features to predictions, but they assume that features are independent. Although we removed highly collinear features during data preprocessing, the potential for prediction interference due to feature correlations cannot be entirely eliminated. This limitation could potentially be addressed through more stringent feature selection, but such an approach may also lead to the loss of more information.

## 5 Conclusion

In conclusion, we developed and validated predictive models for CVD-cancer comorbidity using RPART, RF, K–KNN, NB, and LightGBM. Among these five algorithms, LightGBM demonstrated the highest discriminability and accuracy for predicting CVD-cancer comorbidity. SHAP values clarified the significance and contributions of antioxidants, with naringenin and Mg identified as the primary antioxidants in this model.

## CRediT authorship contribution statement

**Xiangjun Qi:** Writing – review & editing, Writing – original draft, Software, Methodology, Data curation, Conceptualization. **Shujing Wang:** Writing – original draft, Data curation. **Caishan Fang:** Writing – review & editing, Data curation. **Jie Jia:** Writing – review & editing, Data curation. **Lizhu Lin:** Writing – review & editing, Methodology, Data curation, Conceptualization. **Tianhui Yuan:** Writing – review & editing, Methodology, Data curation, Conceptualization.

## Ethics statement

The current study was supported by the Ethics Review Board of U.S. National Center for Health Statistics, and written informed consents were obtained from all participants of the NAHNES survey.

## Availability of data and materials

The datasets used and/or analyzed in the current study are available in the article or supplementary material.

## Consent for publication

Not Applicable.

## Funding information

National Natural Science Foundation of China (82274416); Guangdong Special Support Plan (0720240225); National Key Laboratory of Chinese Medicine Syndrome (SKLKY2024B0020); Guangdong Science and Technology Bureau Key Research and Development Plan (SL2022B01J10007); National Key Research and Development Program of the Ministry of Science and Technology (2022YFC3500203); Basic Research Program of GuangZhou Science and Technology Bureau (2023A03J0300); Basic and Applied Basic Research Foundation of Guangdong Province (2023A03J0300).

## Declaration of competing interest

The authors report no conflicts of interest in this work.

## Data Availability

Data will be made available on request.
