# Supplementary material for: Machine learning and SHAP value interpretation for predicting comorbidity of cardiovascular disease and cancer with dietary antioxidants
Source: Redox Biol. 2024 Dec 16;79:103470. doi: 10.1016/j.redox.2024.103470 (PMC11729017; doi:10.1016/j.redox.2024.103470)
Supplement: Multimedia component 1 [file mmc1.docx]

Supplementary Table 1. Baseline characteristics of the participants.

|  | Nom-Comorbid CVD and cancer | Comorbid CVD and cancer | *p*-value |
| --- | --- | --- | --- |
| Vitamin A | 644.895( 9.798) | 643.935(30.069) | 0.972 |
| Vitamin C | 81.144(1.292) | 76.105(3.748) | 0.161 |
| Vitamin E | 8.392(0.094) | 7.797(0.282) | 0.051 |
| Mg | 302.756(2.976) | 260.893(6.131) | < 0.0001 |
| Zinc | 11.765(0.133) | 10.489(0.328) | < 0.001 |
| Se | 114.166(0.835) | 97.070(3.122) | < 0.0001 |
| Carotenoid | 9757.494(200.947) | 9982.854(704.515) | 0.753 |
| Daidzein | 0.833(0.053) | 0.695(0.289) | 0.637 |
| Genistein | 1.180(0.077) | 0.913(0.389) | 0.503 |
| Glycitein | 0.172(0.012) | 0.138(0.067) | 0.614 |
| Cyanidin | 2.651(0.188) | 2.483(0.362) | 0.677 |
| Petunidin | 1.202(0.108) | 1.419(0.289) | 0.457 |
| Delphinidin | 1.713(0.153) | 1.725(0.333) | 0.974 |
| Malvidin | 4.842(0.318) | 7.197(1.166) | 0.05 |
| Pelargonidin | 1.551(0.101) | 1.954(0.413) | 0.327 |
| Peonidin | 2.158(0.205) | 2.814(0.993) | 0.546 |
| Catechin | 7.743(0.168) | 7.752(0.683) | 0.989 |
| Epigallocatechin | 16.327(0.721) | 16.218(3.941) | 0.978 |
| Epicatechin | 9.985(0.210) | 9.338(1.083) | 0.553 |
| Epicatechin 3 gallate | 10.536(0.473) | 10.777(2.694) | 0.929 |
| Epigallocatechin 3 gallate | 27.871(1.335) | 26.450(6.689) | 0.832 |
| Theaflavin | 1.583(0.076) | 1.873(0.472) | 0.543 |
| Thearubigins | 90.894( 4.090) | 106.380(25.030) | 0.536 |
| Eriodictyol | 0.189(0.012) | 0.128(0.039) | 0.083 |
| Hesperetin | 8.695(0.280) | 7.267(0.777) | 0.076 |
| Naringenin | 3.290(0.170) | 4.338(1.486) | 0.486 |
| Apigenin | 0.211(0.015) | 0.207(0.024) | 0.887 |
| Luteolin | 0.708(0.020) | 0.709(0.061) | 0.987 |
| Isorhamnetin | 0.888(0.023) | 0.730(0.067) | 0.014 |
| Kaempferol | 4.848(0.105) | 3.904(0.515) | 0.066 |
| Myricetin | 1.538(0.045) | 1.550(0.211) | 0.954 |
| Quercetin | 11.477(0.188) | 10.965(0.877) | 0.544 |
| Theaflavin 3-3 digallate | 1.744(0.085) | 2.072(0.521) | 0.535 |
| Theaflavin 3q gallate | 1.481(0.072) | 1.756(0.447) | 0.544 |
| Theaflavin 3 gallate | 1.252(0.061) | 1.495(0.374) | 0.521 |
| Gallocatechin | 1.702(0.073) | 1.793(0.448) | 0.84 |
| Subtotal Catechins | 74.164( 2.857) | 72.327(15.287) | 0.905 |
| Total Isoflavones | 2.185(0.141) | 1.746(0.743) | 0.562 |
| Total Anthocyanidins | 14.117(0.789) | 17.591(2.326) | 0.166 |
| Total Flavan 3 ols | 171.119( 6.916) | 185.902(41.779) | 0.723 |
| Total Flavanones | 12.174(0.420) | 11.734(1.786) | 0.808 |
| Total Flavones | 0.919(0.027) | 0.916(0.079) | 0.97 |
| Total Flavonols | 18.751(0.327) | 17.149(1.564) | 0.292 |
| Total Sum of all 29 flavonoids | 219.265( 7.221) | 235.039(42.858) | 0.711 |
| Age |  |  | < 0.0001 |
| <65 | 8080(88.082) | 84(27.391) |  |
| >=65 | 1631(11.918) | 269(72.609) |  |
| Gender |  |  | 0.128 |
| Female | 5112(51.869) | 152(47.087) |  |
| Male | 4599(48.131) | 201(52.913) |  |
| BMI level |  |  | 0.012 |
| Underweight | 153(1.660) | 4(0.784) |  |
| Normal weight | 2522(27.992) | 73(19.605) |  |
| Overweight | 3178(32.291) | 120(32.334) |  |
| Obesity | 3858(38.057) | 156(47.277) |  |
| Race |  |  | 0.002 |
| Mexican American | 1650(8.612) | 16(1.671) |  |
| Non-Hispanic Black | 2035(11.298) | 53( 6.870) |  |
| Non-Hispanic White | 4188(67.319) | 265(85.669) |  |
| Other Hispanic | 988(5.394) | 8(1.178) |  |
| Other Race - Including Multi-Racial | 850(7.377) | 11(4.611) |  |
| Education |  |  | 0.016 |
| College or above | 2210(29.890) | 59(22.842) |  |
| High school graduate | 2288(24.692) | 98(31.373) |  |
| Less than high school | 2289(14.537) | 102(19.657) |  |
| Some college or associates degree | 2924(30.881) | 94(26.129) |  |
| Diabetes |  |  | < 0.0001 |
| No | 8203(89.074) | 204(56.441) |  |
| Yes | 1508(10.926) | 149(43.559) |  |
| Hyperlipidemia |  |  | < 0.0001 |
| No | 3019(32.739) | 32( 8.886) |  |
| Yes | 6692(67.261) | 321(91.114) |  |
| Hypertension |  |  | < 0.0001 |
| No | 6119(67.903) | 57(16.188) |  |
| Yes | 3592(32.097) | 296(83.812) |  |
| FPIR level |  |  | 0.257 |
| >=0,<=1 | 1957(13.042) | 65(11.283) |  |
| >1,<=3 | 4135(35.644) | 168(41.418) |  |
| >3 | 3619(51.314) | 120(47.299) |  |
| Moderate to vigorous |  |  | < 0.0001 |
| No | 2981(24.367) | 176(46.300) |  |
| Yes | 6730(75.633) | 177(53.700) |  |
| Smoker |  |  | < 0.0001 |
| Former | 2148(22.931) | 178(50.354) |  |
| Never | 5530(57.676) | 117(34.825) |  |
| Current | 2033(19.394) | 58(14.821) |  |
