# Supplementary figures and images for: Machine learning and SHAP value interpretation for predicting comorbidity of cardiovascular disease and cancer with dietary antioxidants
Source: Redox Biol. 2024 Dec 16;79:103470. doi: 10.1016/j.redox.2024.103470 (PMC11729017; doi:10.1016/j.redox.2024.103470)

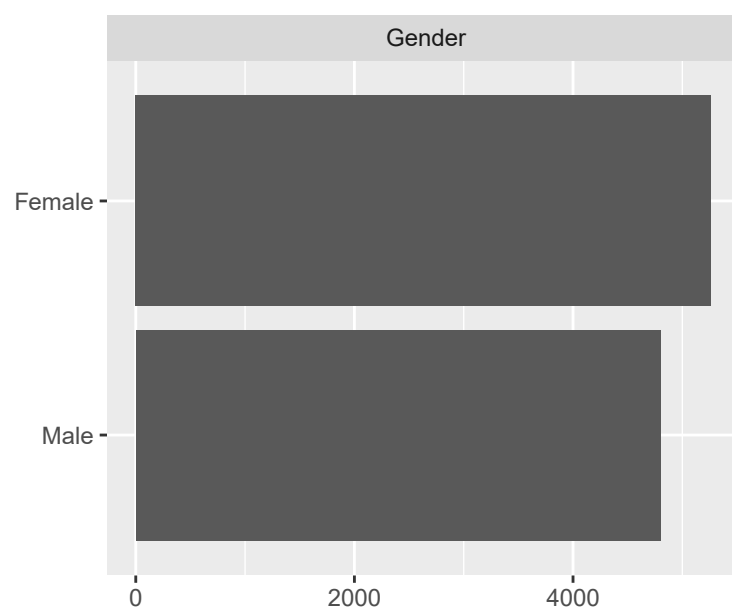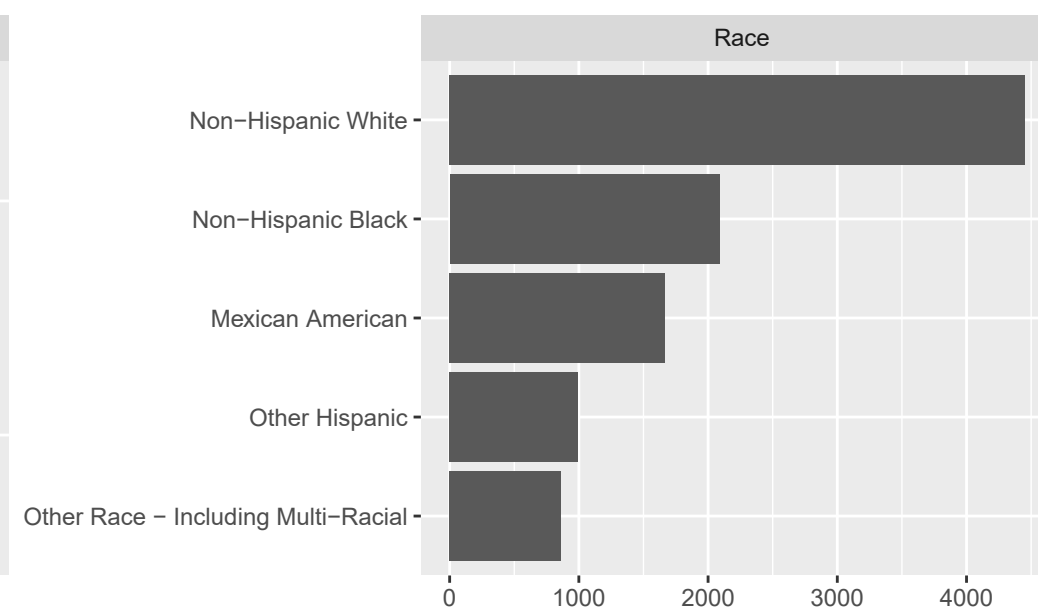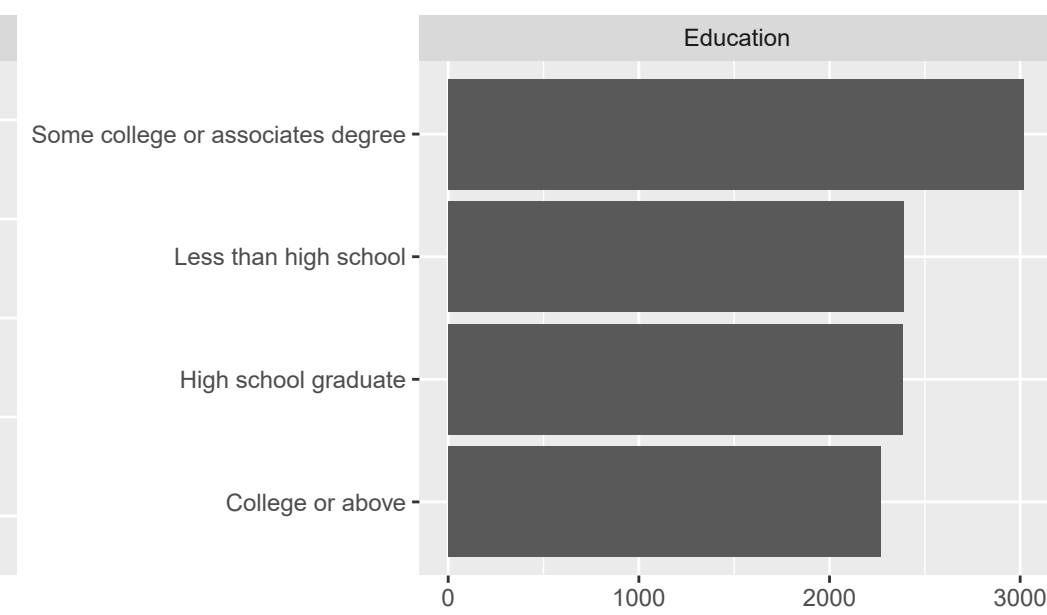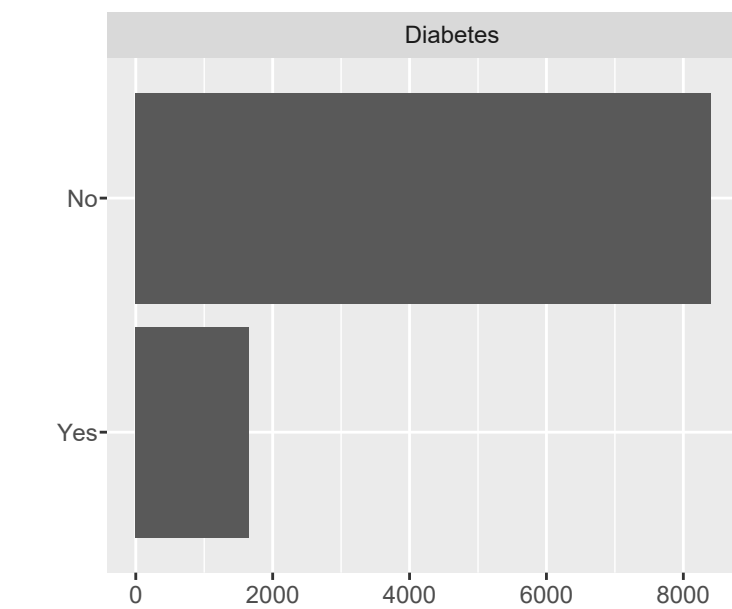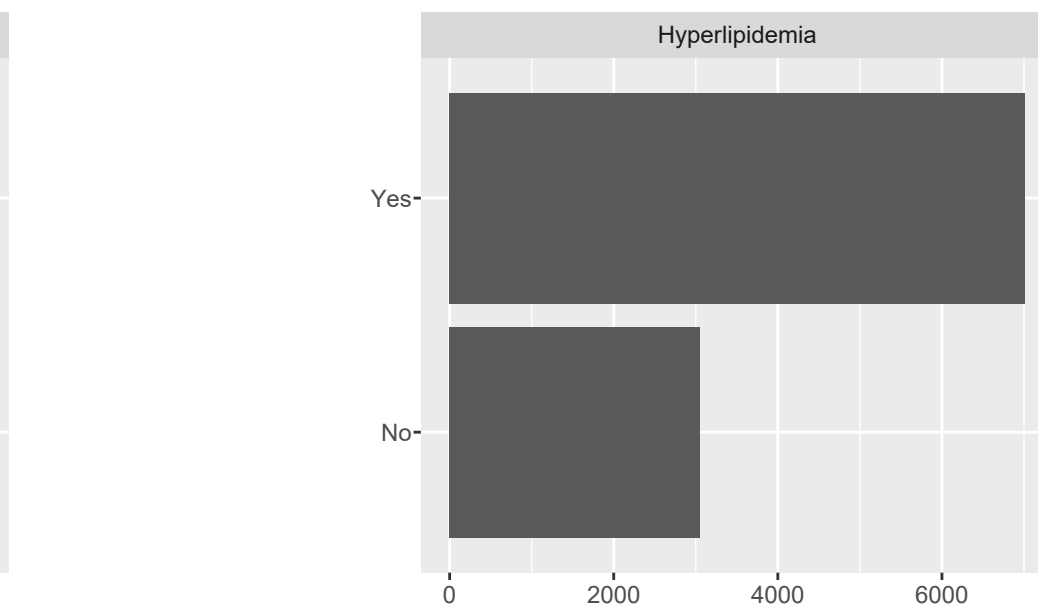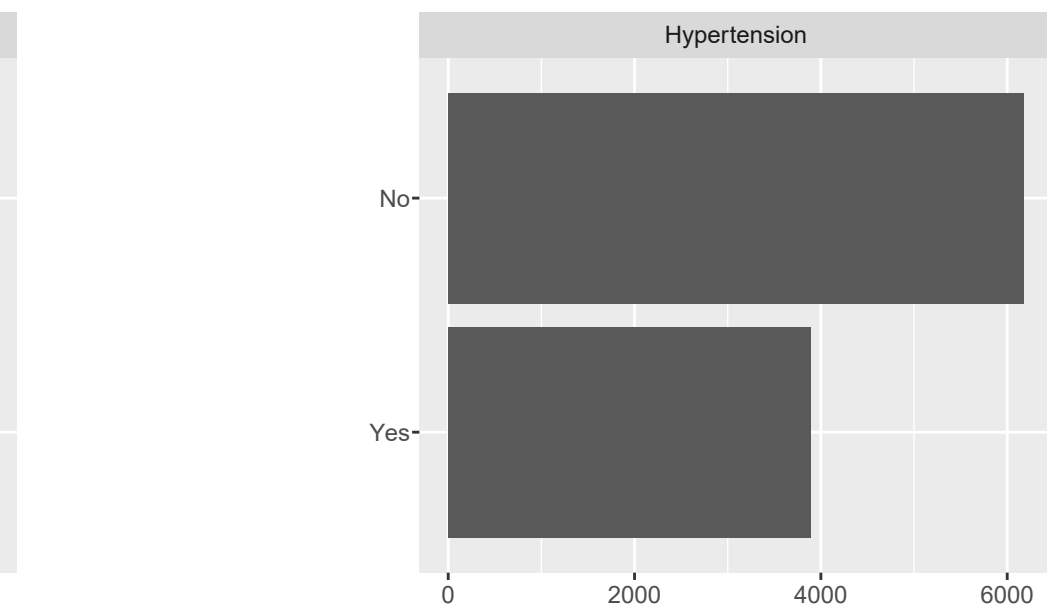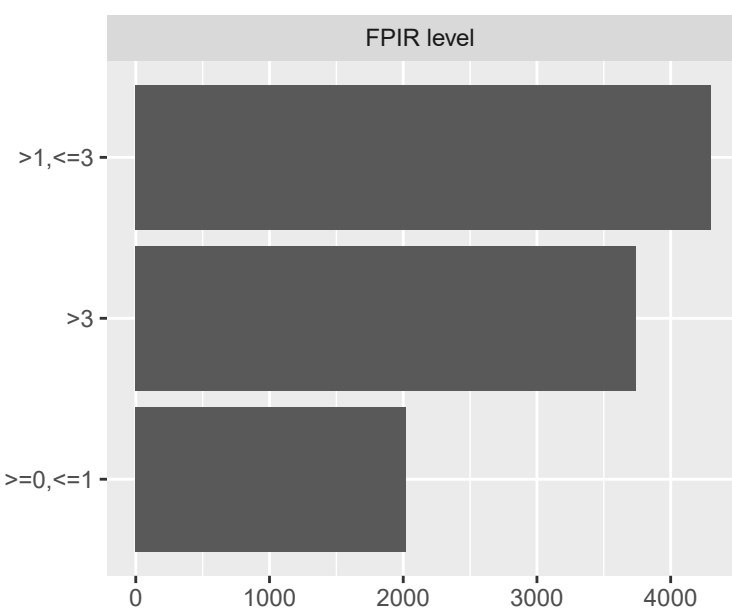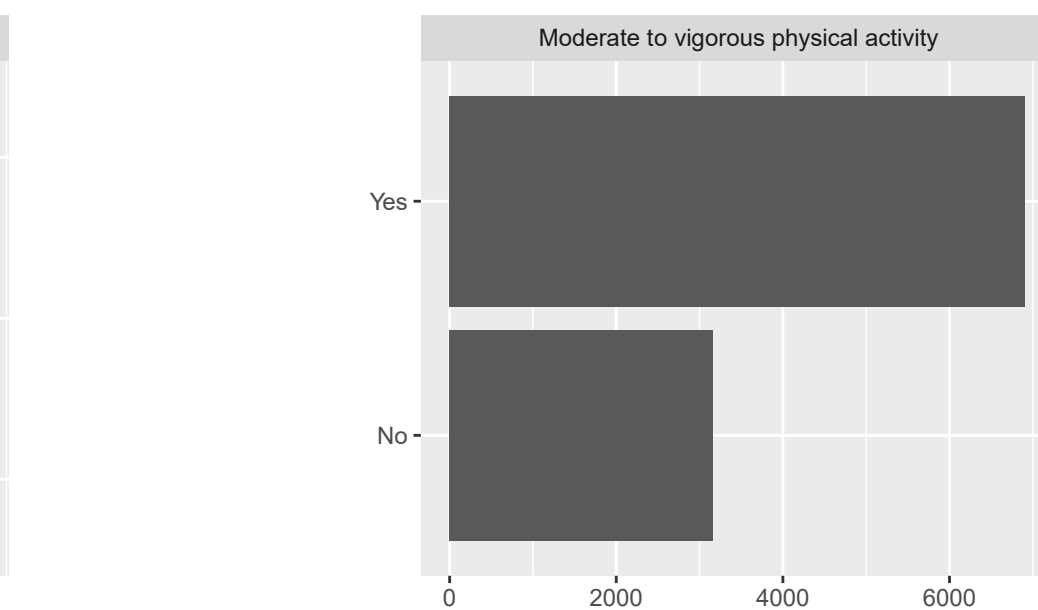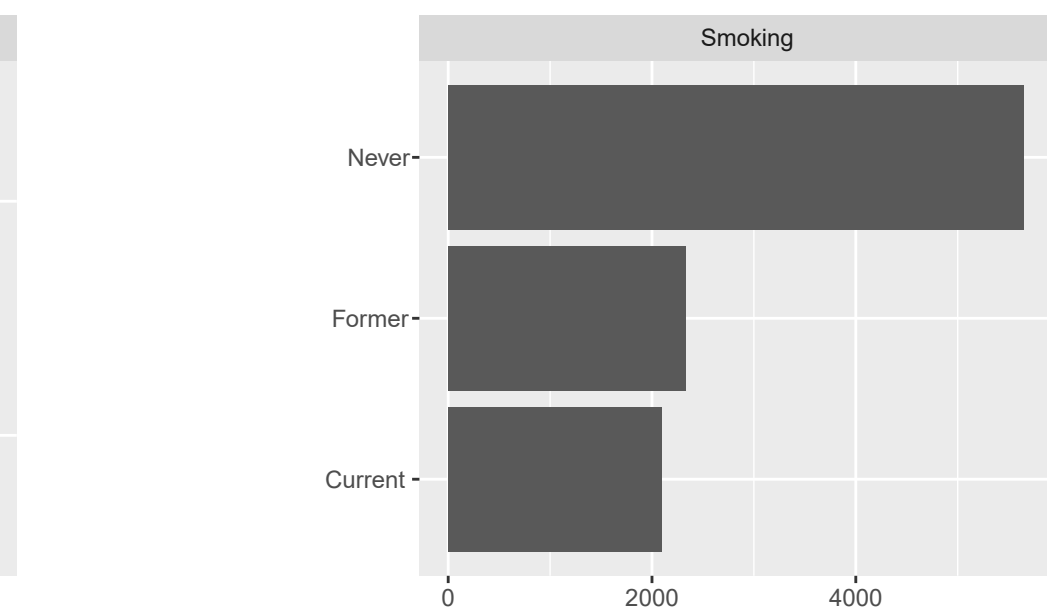

Frequency

Supplement: Multimedia component 2 [file mmc2.pdf]

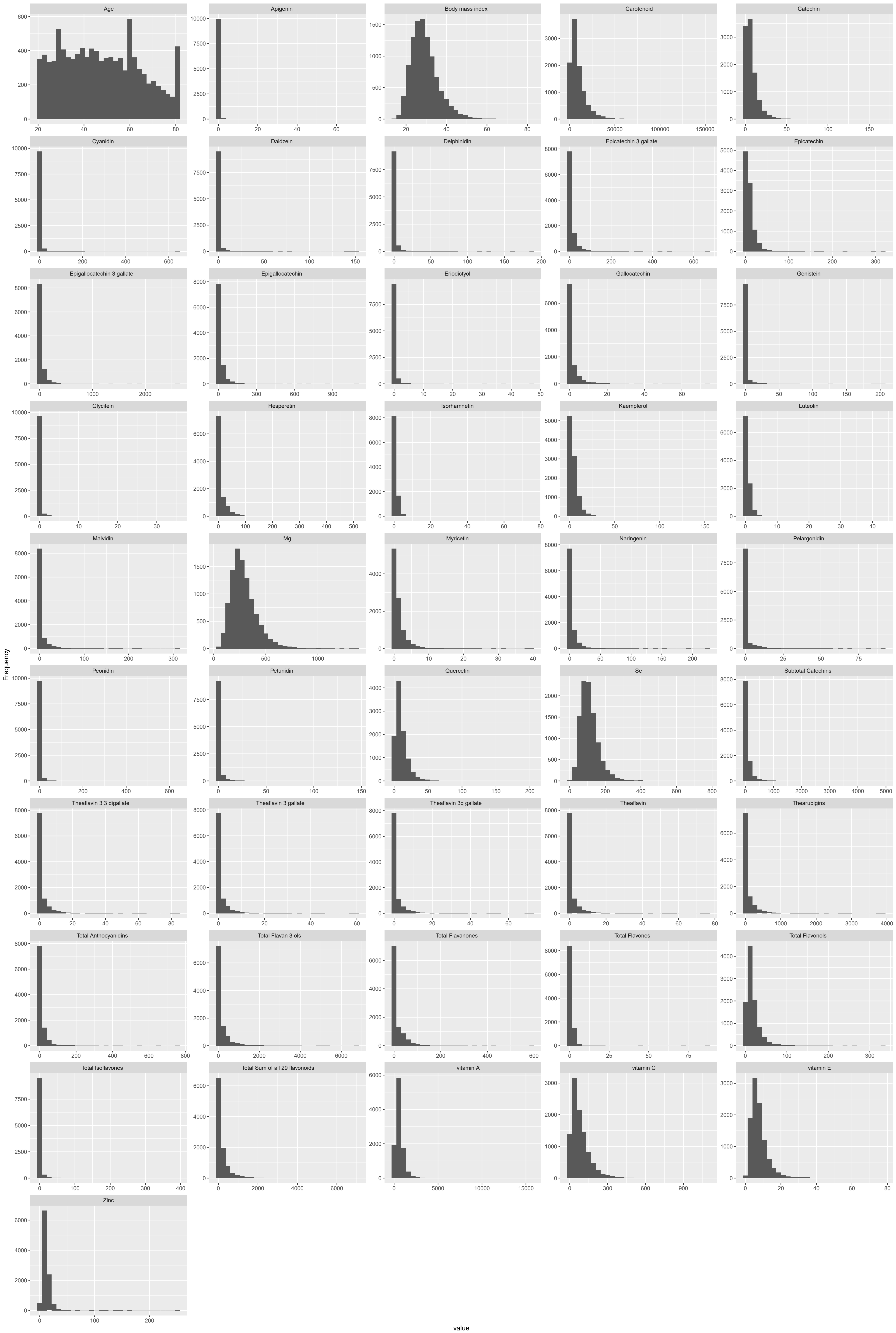

Supplement: Multimedia component 3 [file mmc3.pdf]

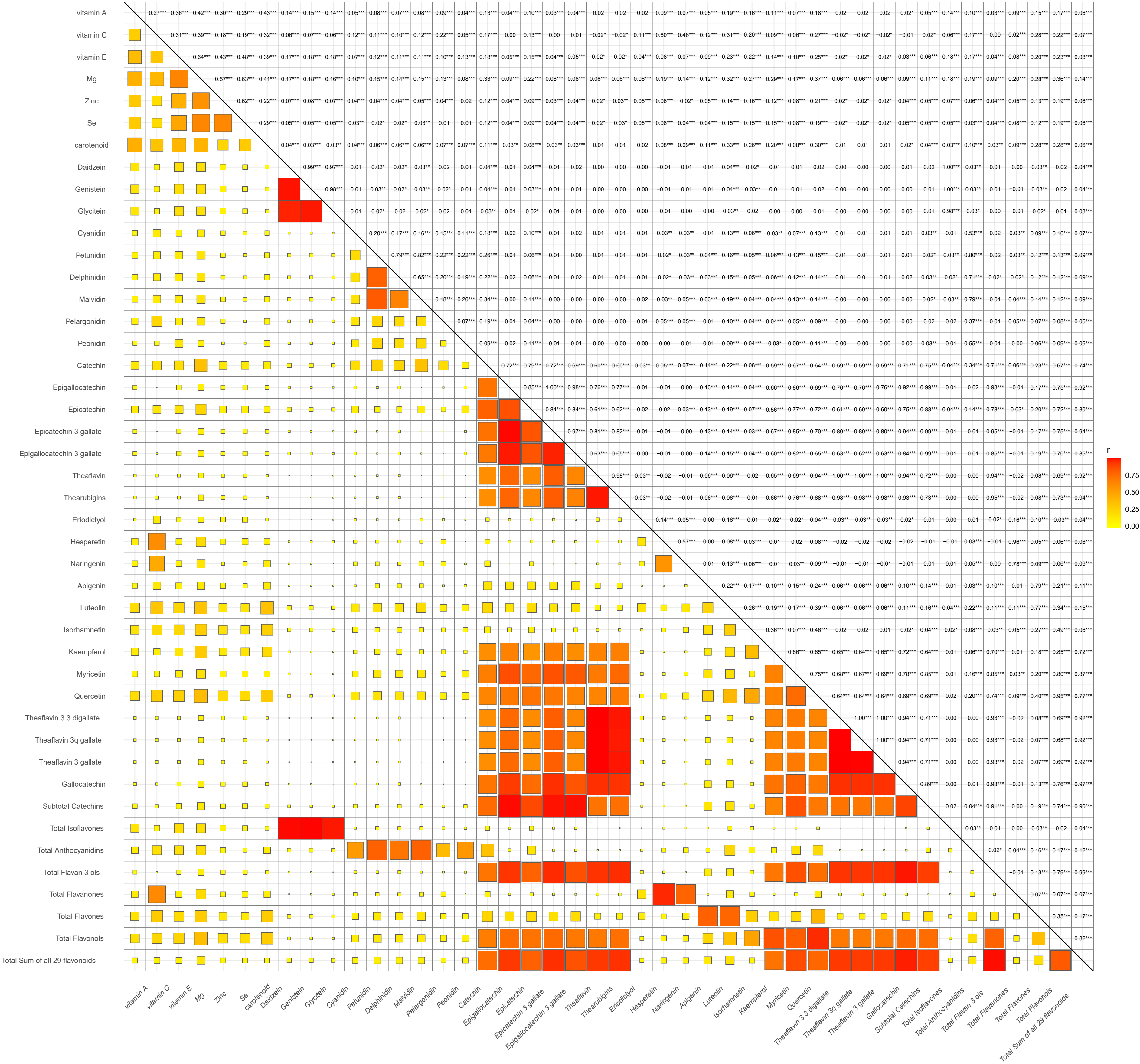

Supplement: Multimedia component 4 [file mmc4.pdf]

Specificity

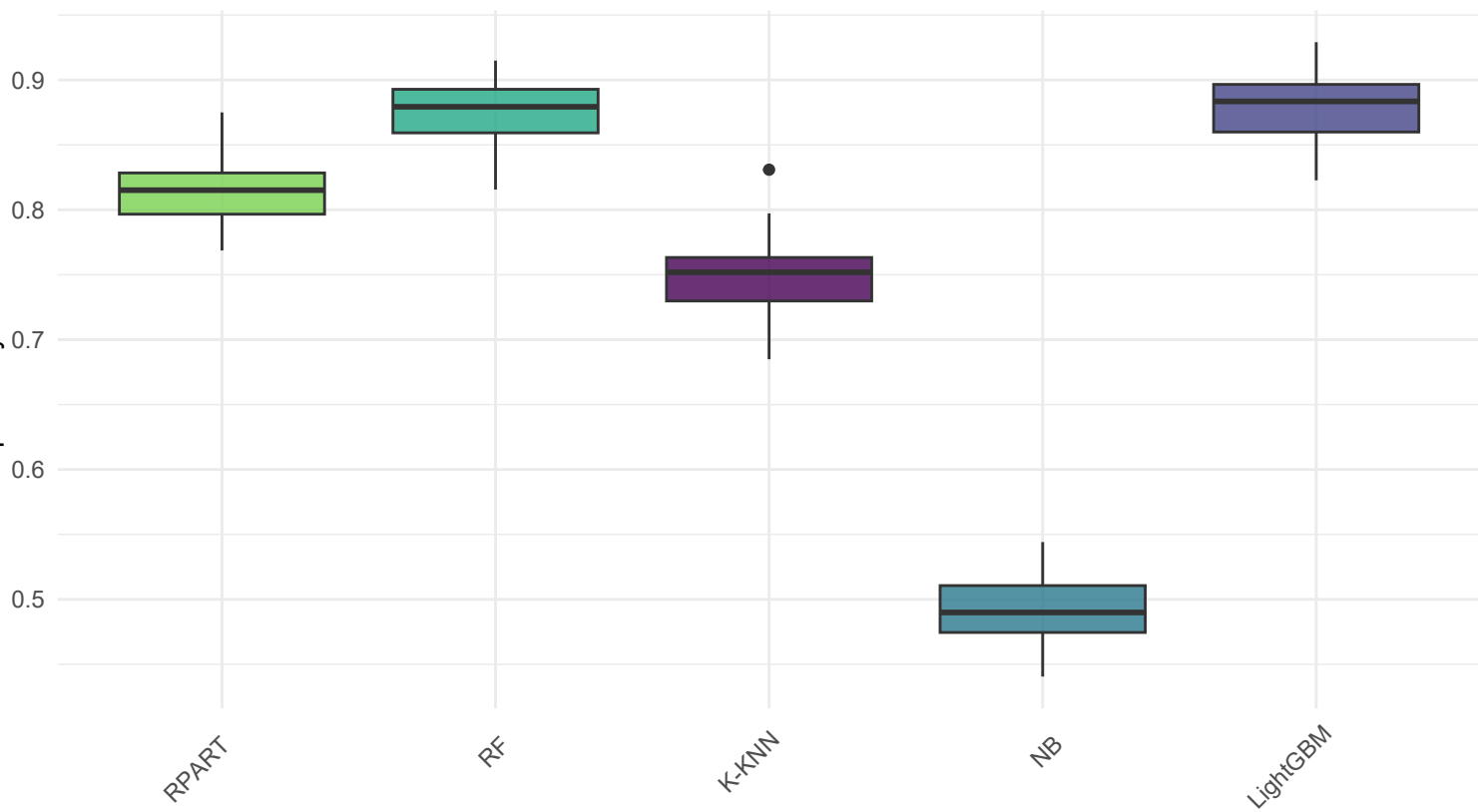

Supplement: Multimedia component 5 [file mmc5.pdf]

Sensitivity

0.95

0.90

0.85

0.80

0.75

Rpart

RF

K-KNN

NB

LightGBM

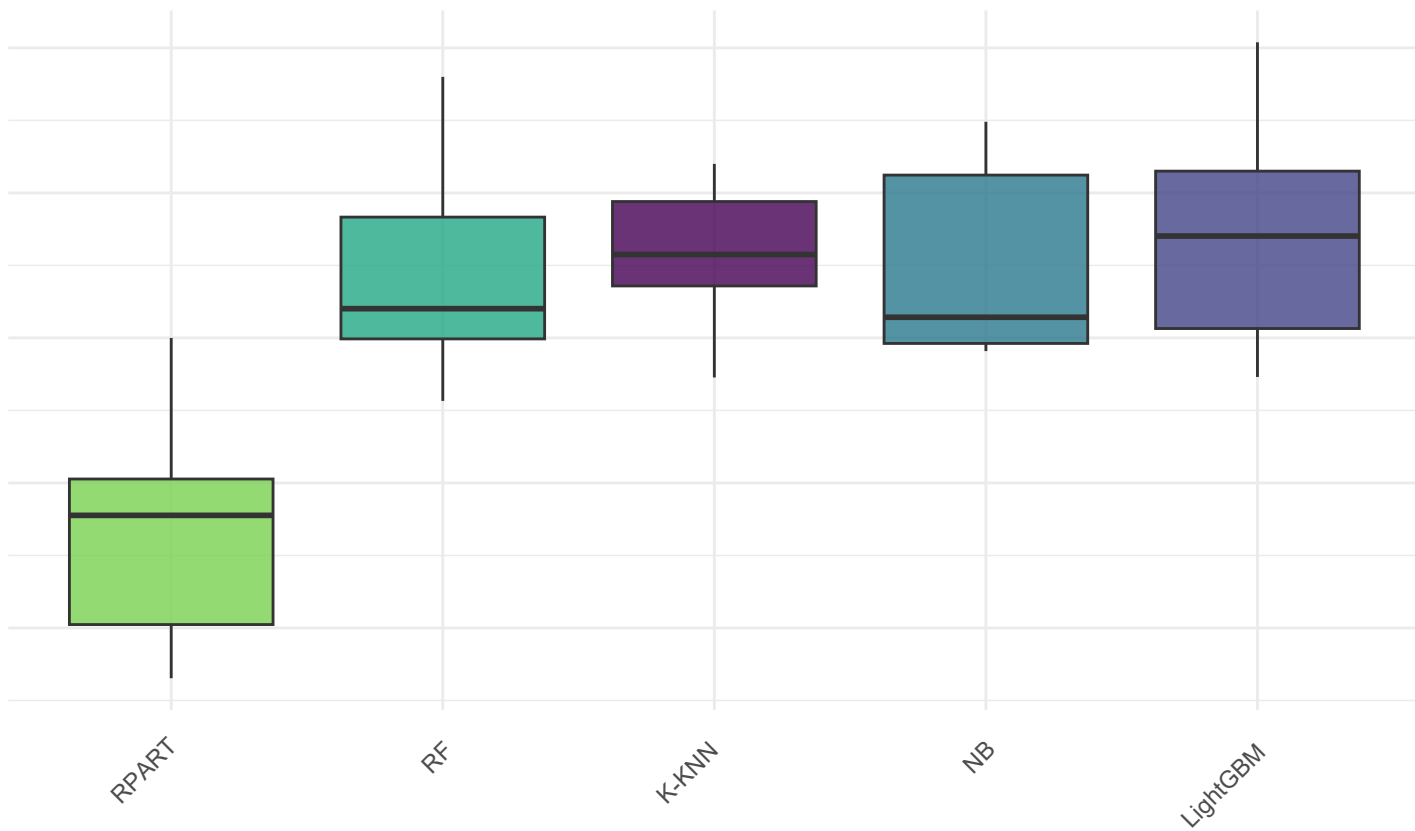

Supplement: Multimedia component 6 [file mmc6.pdf]

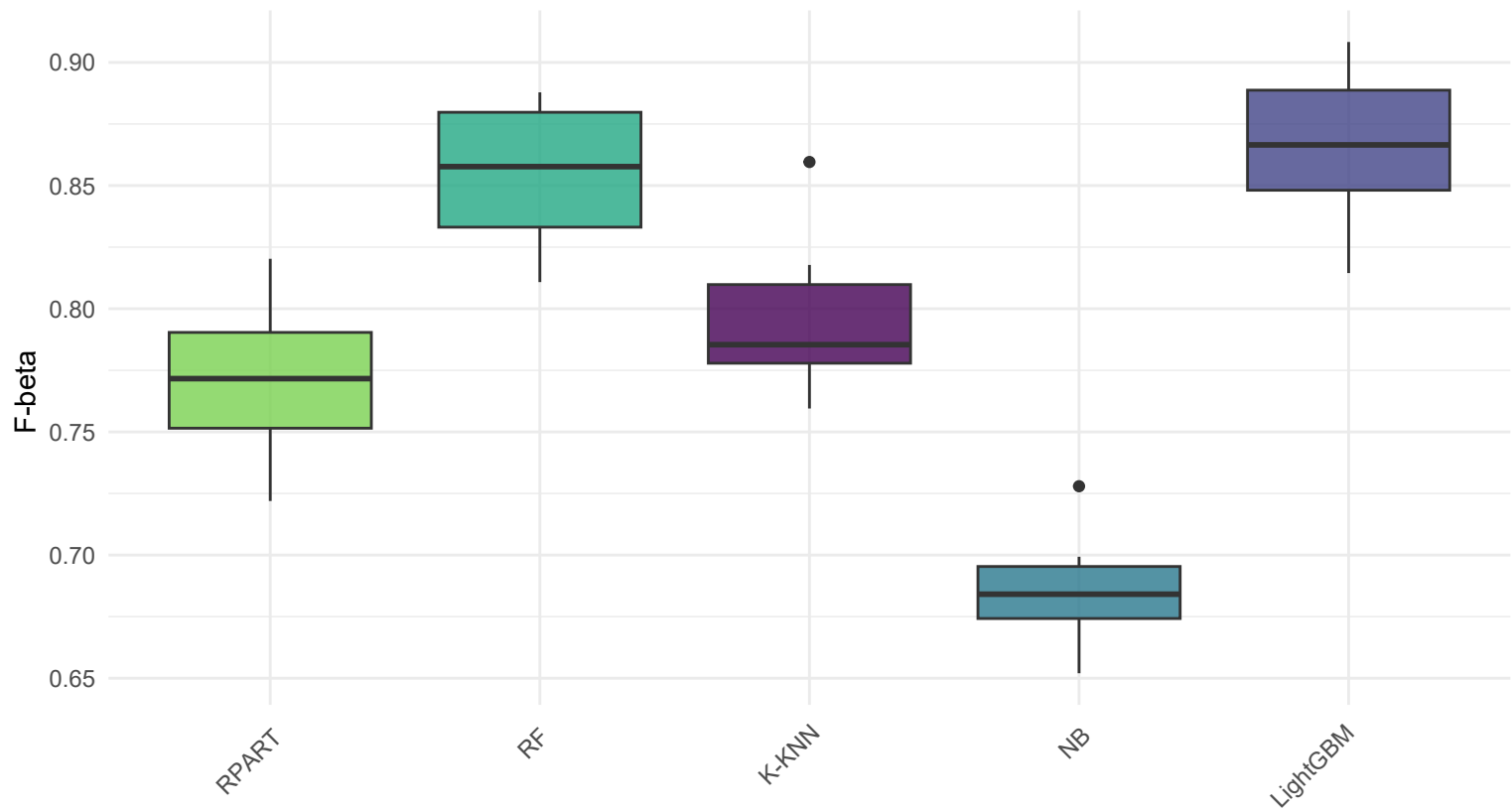

Supplement: Multimedia component 7 [file mmc7.pdf]

Accuracy

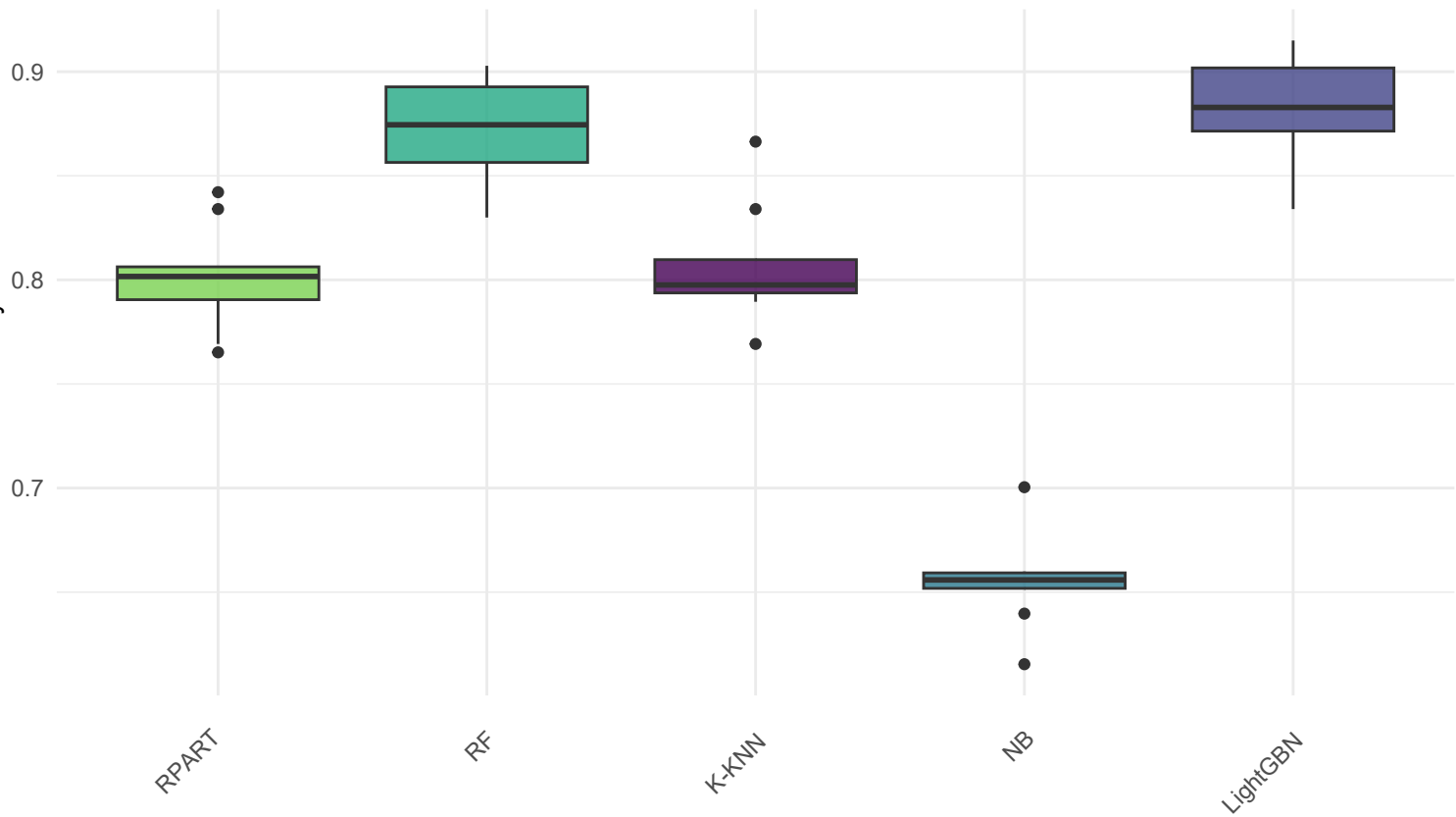

Supplement: Multimedia component 8 [file mmc8.pdf]

Error rate

0.3

0.2

0.1

Rpart

RF

K-knn

NB

LightGBM

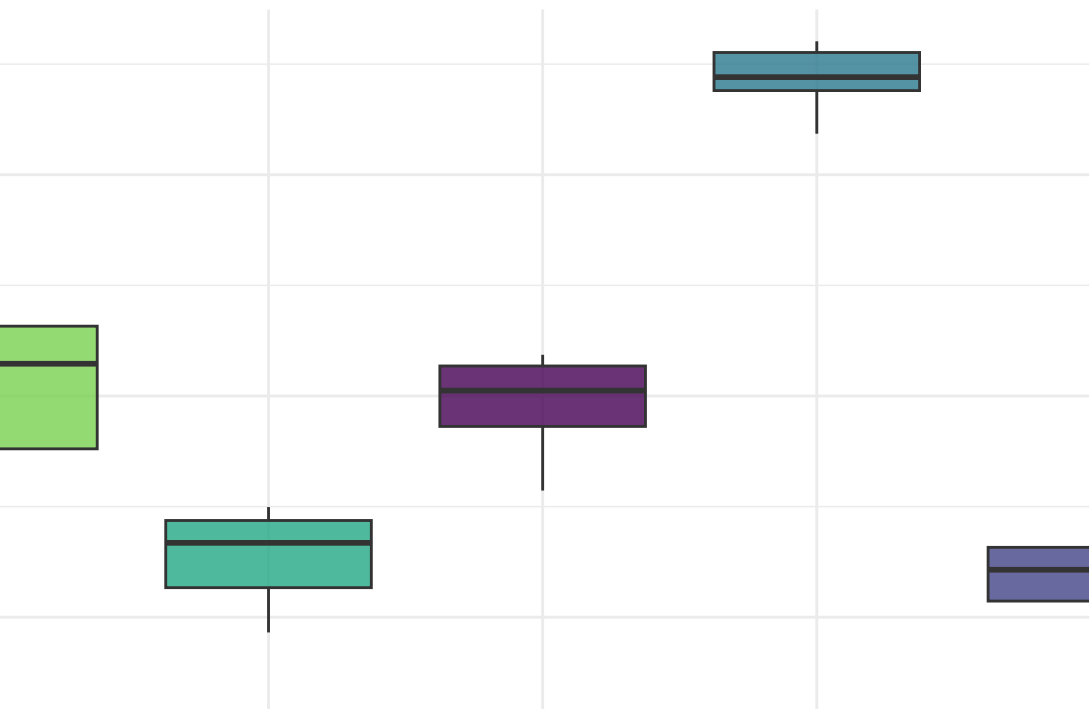

Supplement: Multimedia component 9 [file mmc9.pdf]

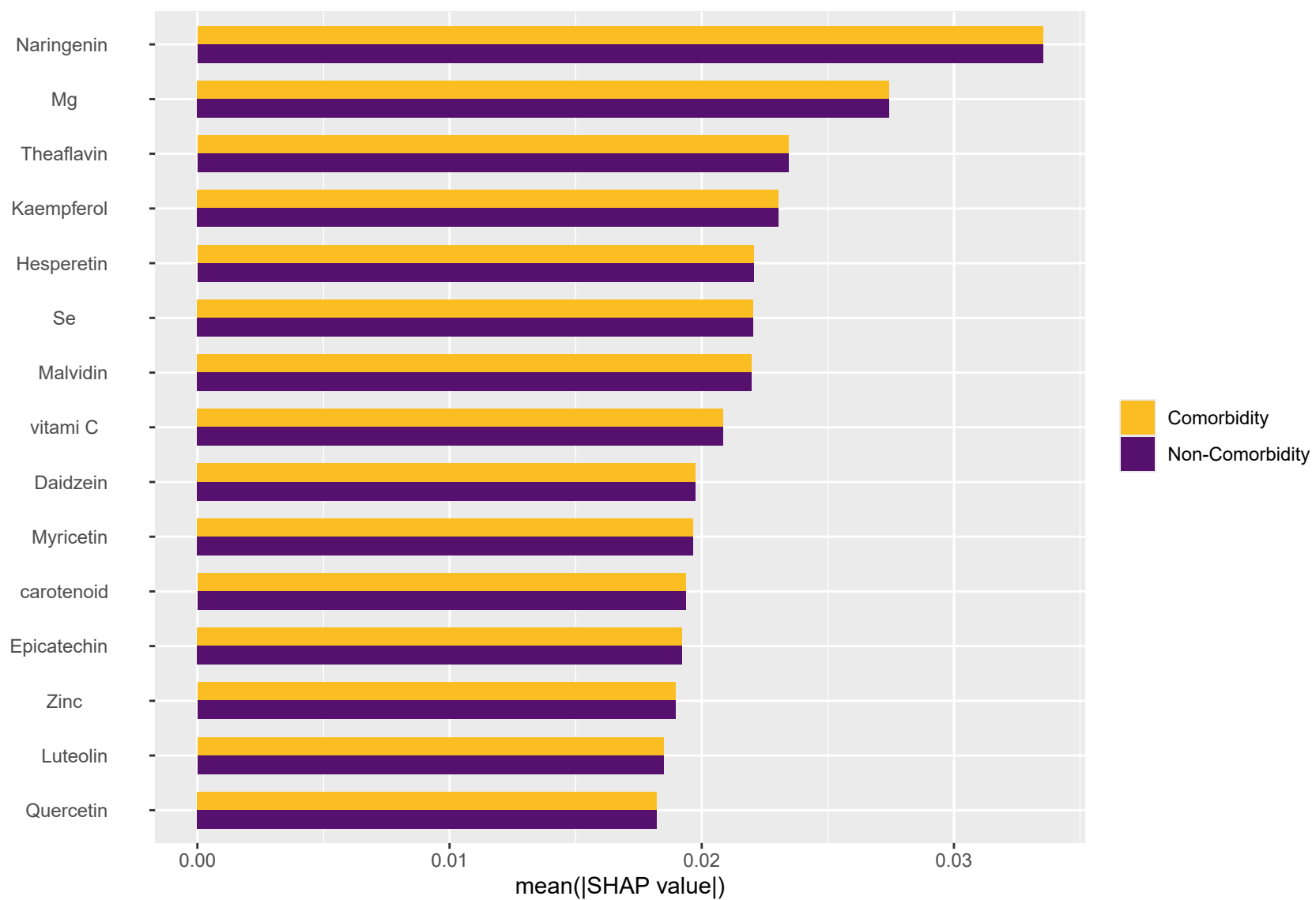

Supplement: Multimedia component 10 [file mmc10.pdf]

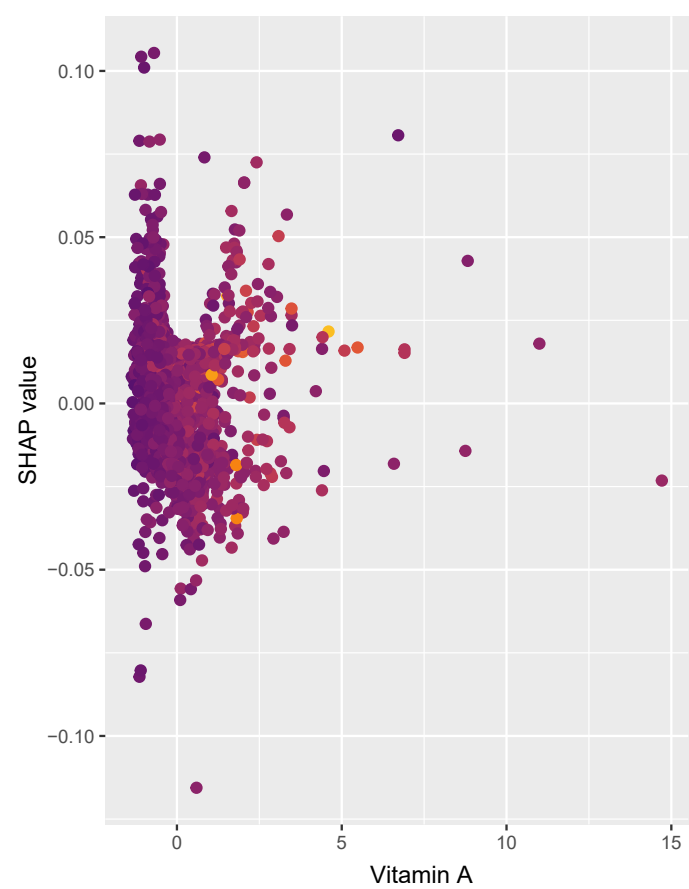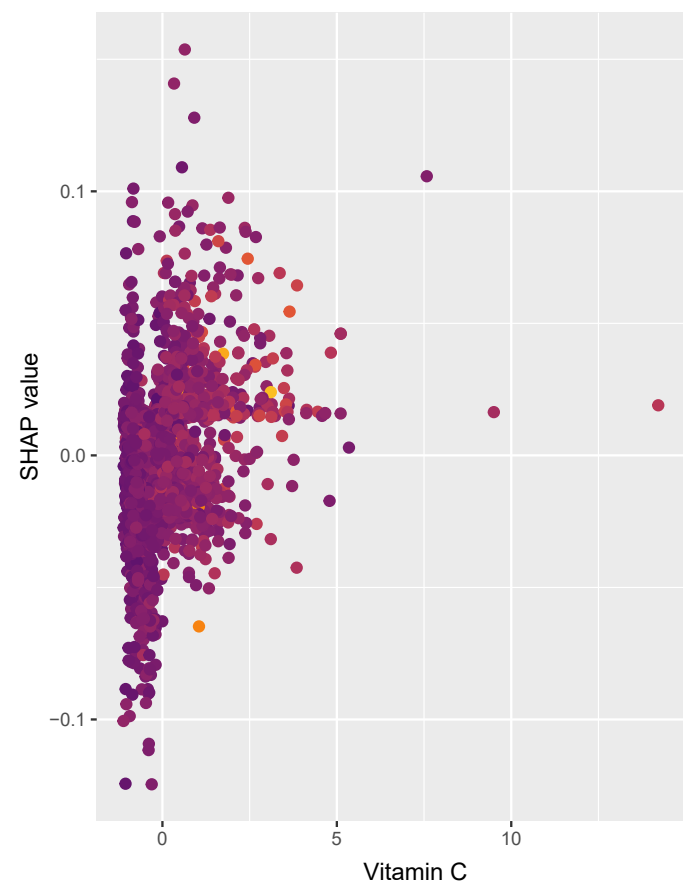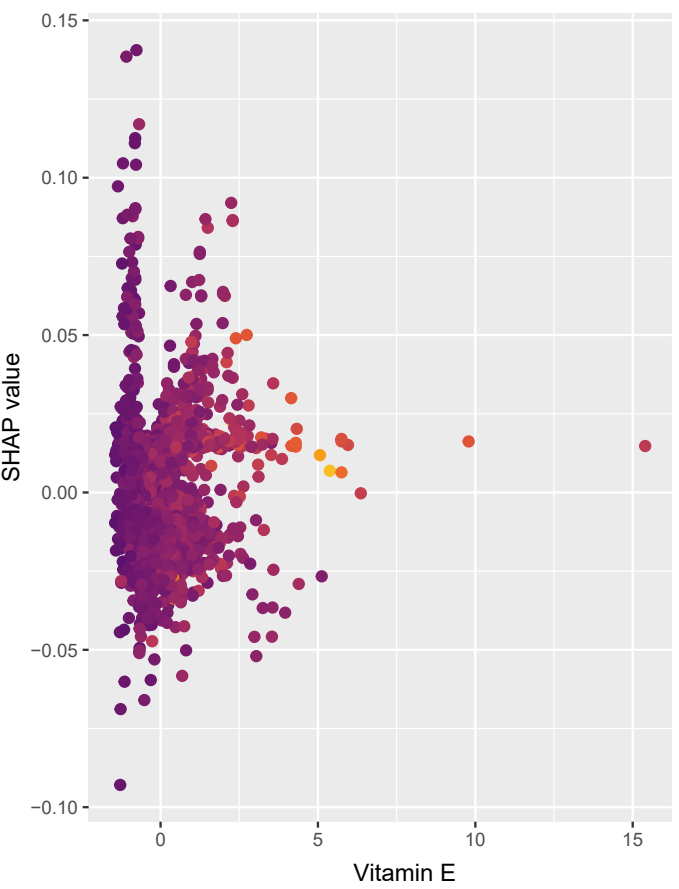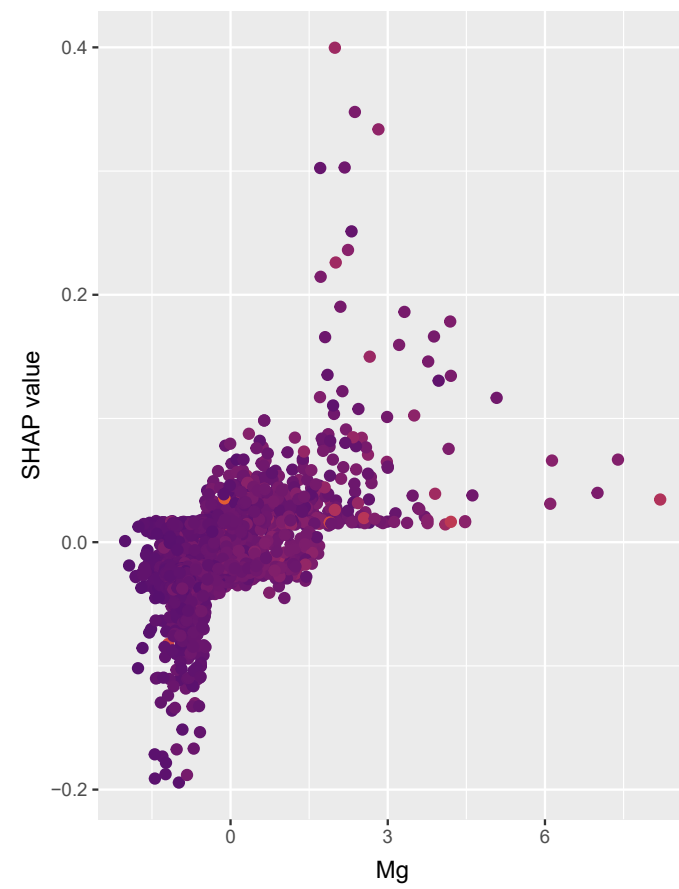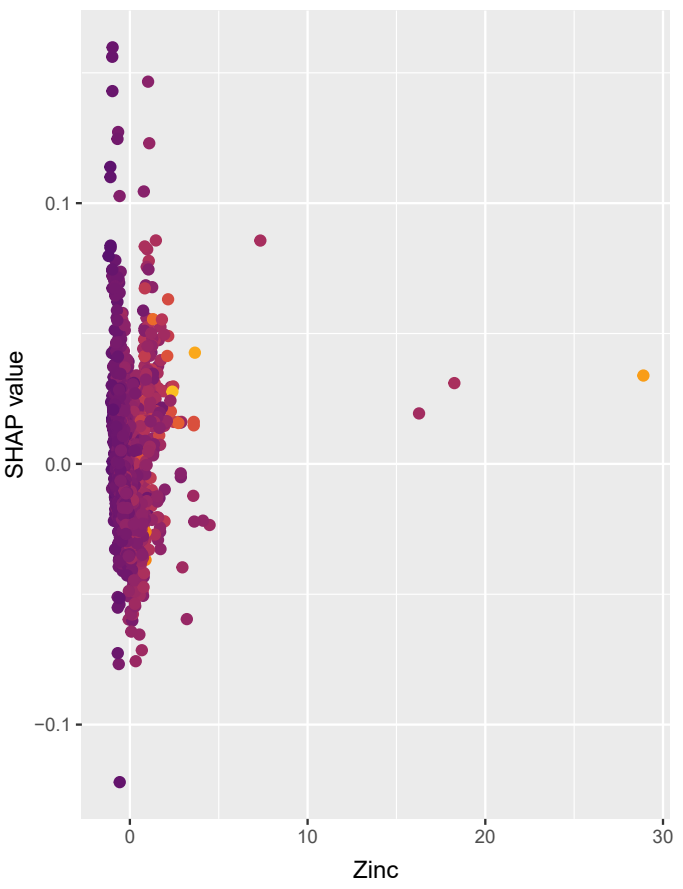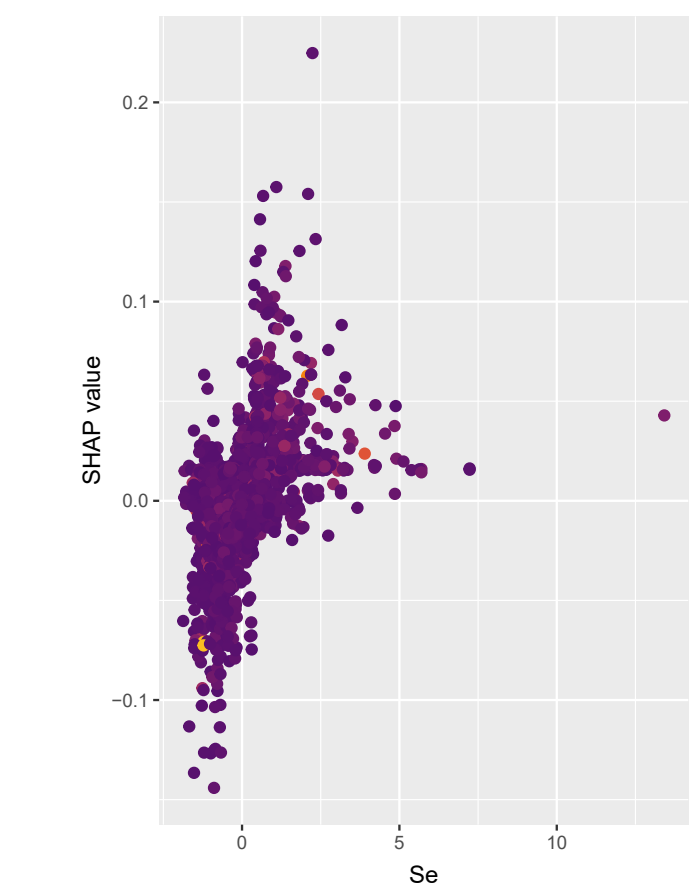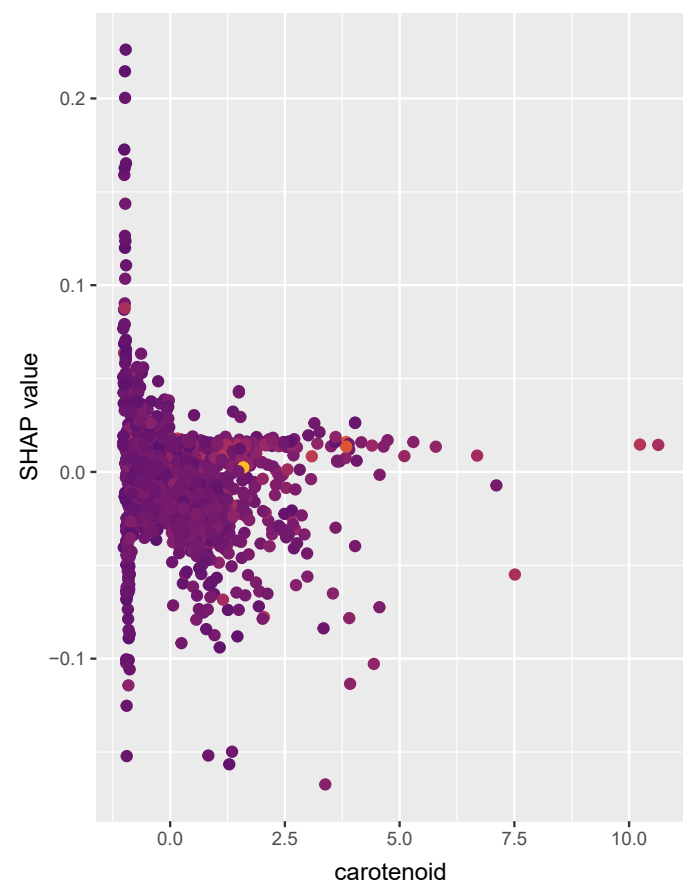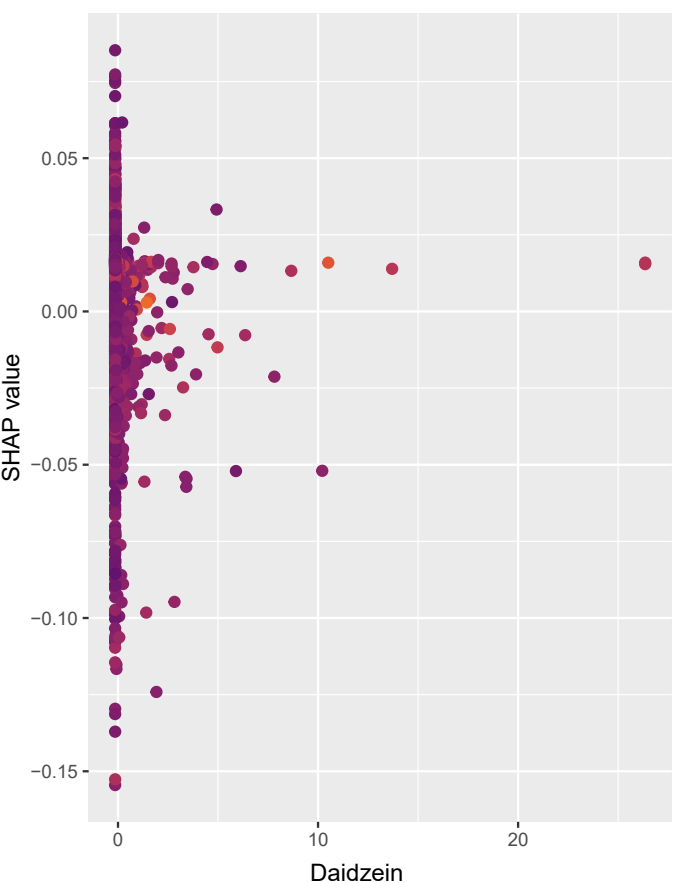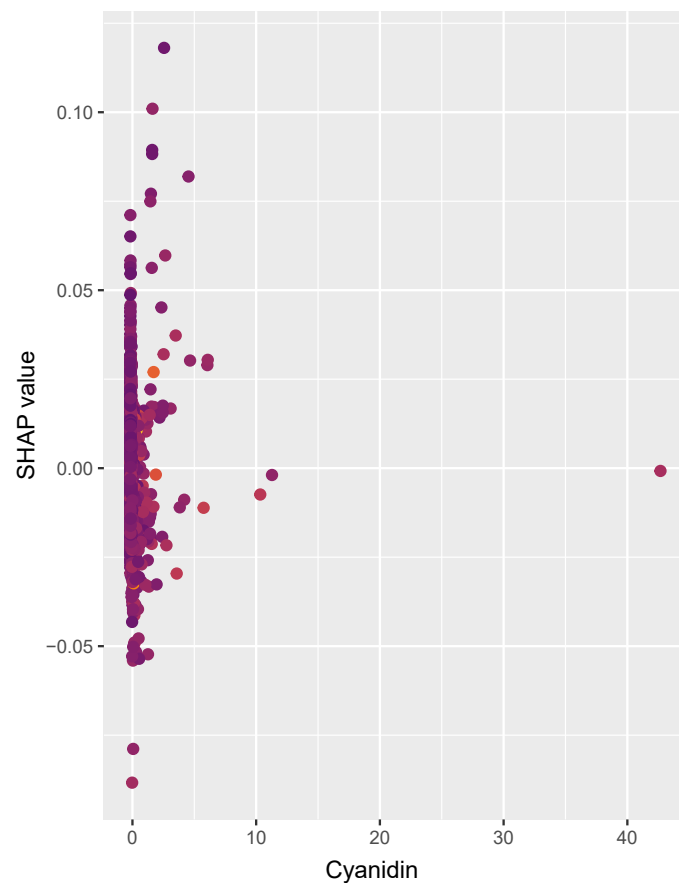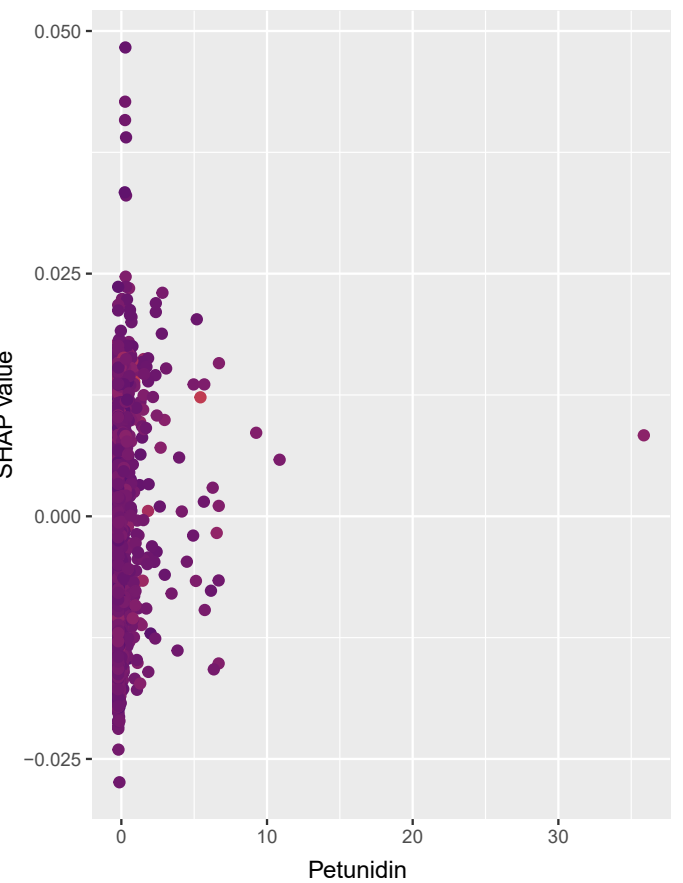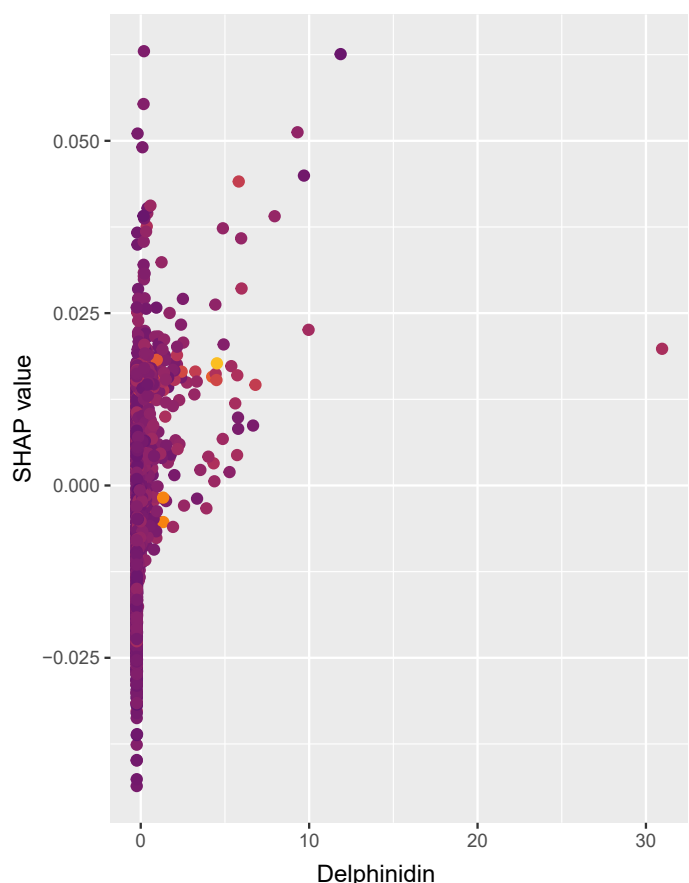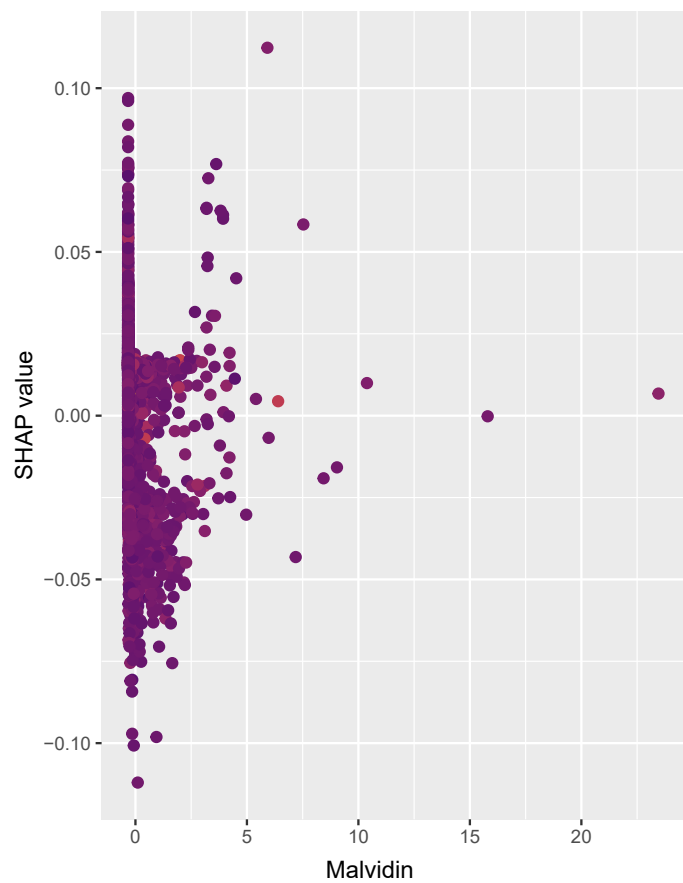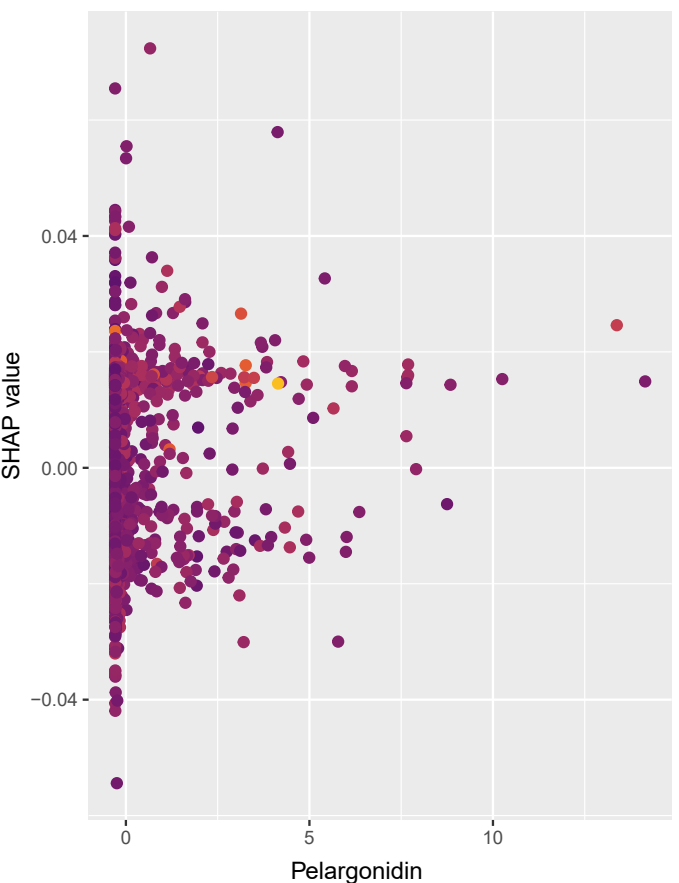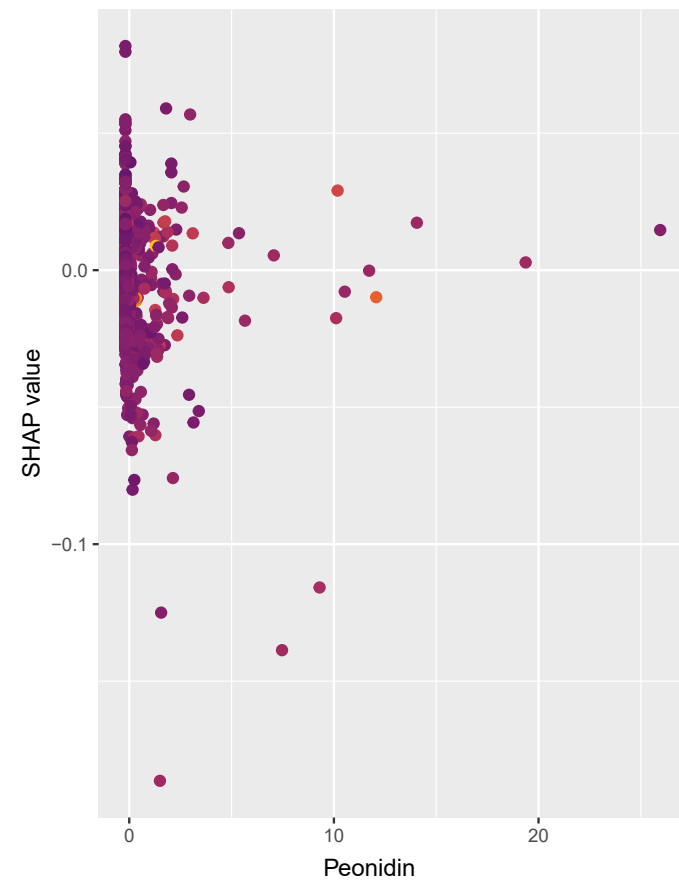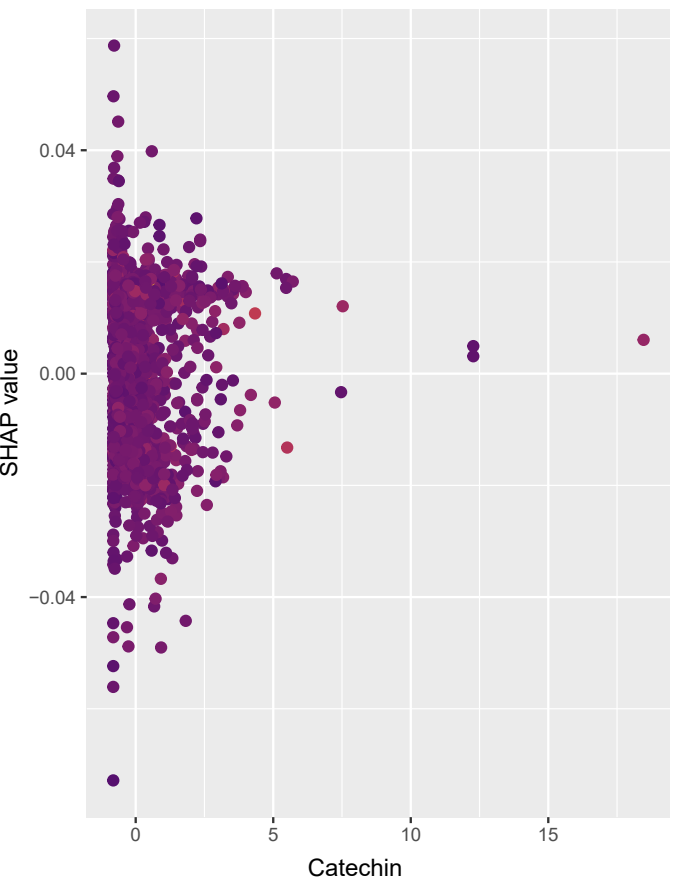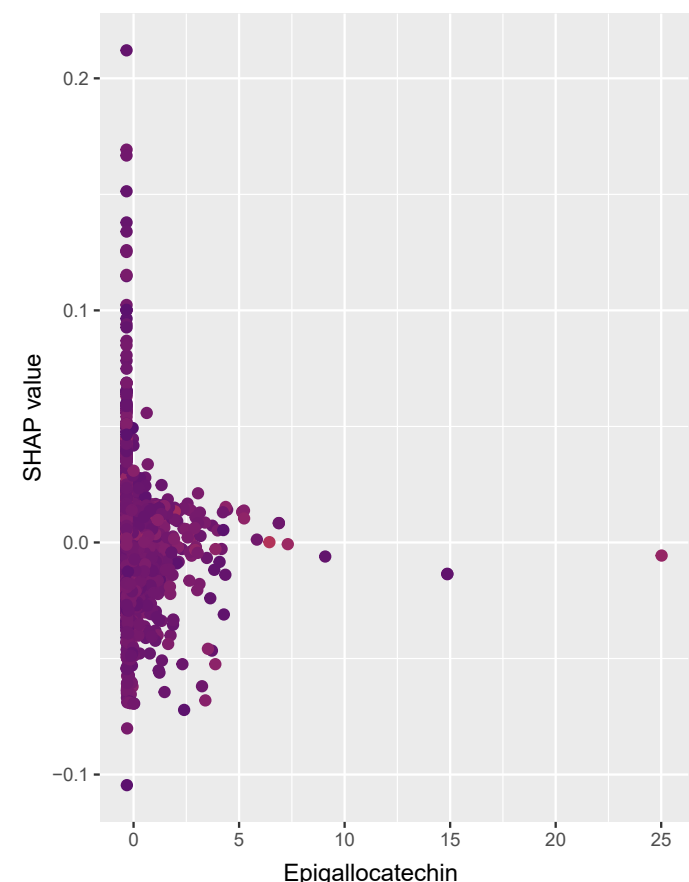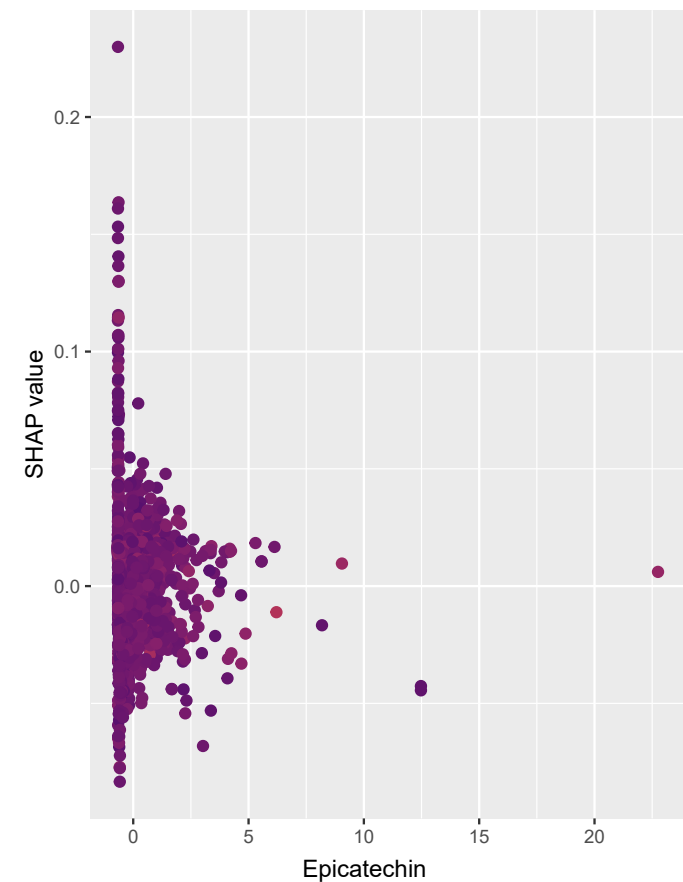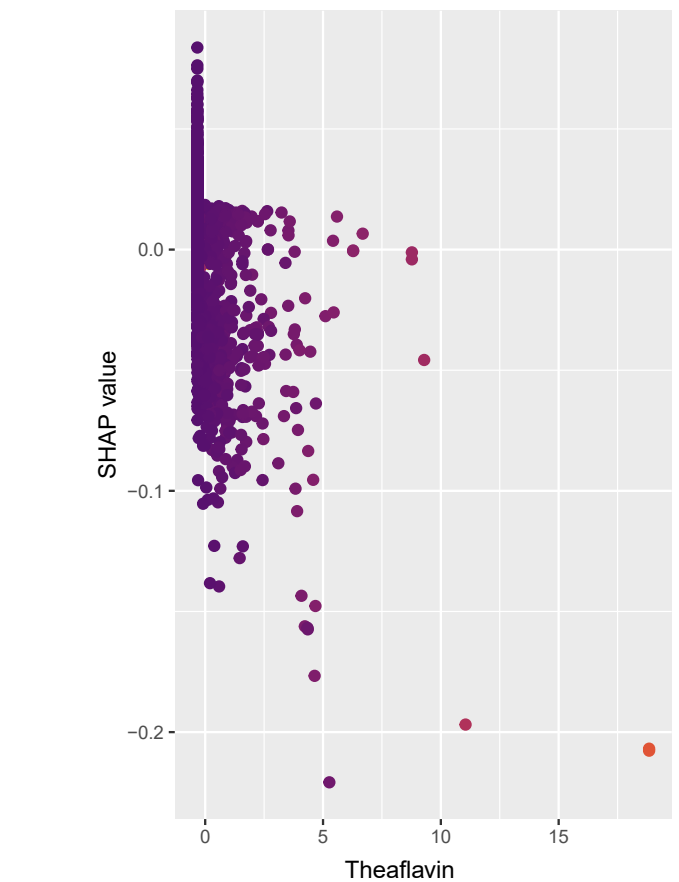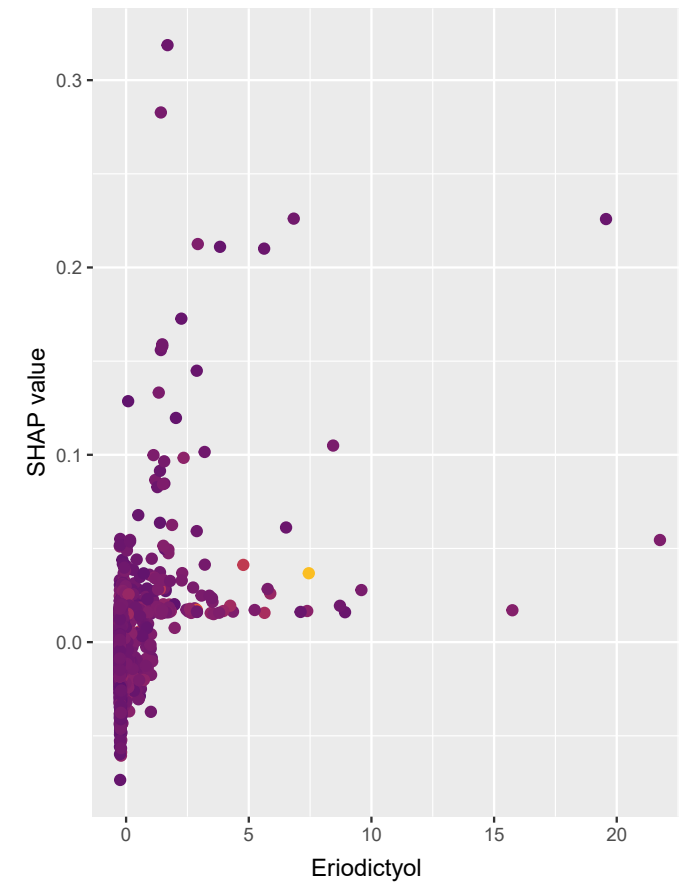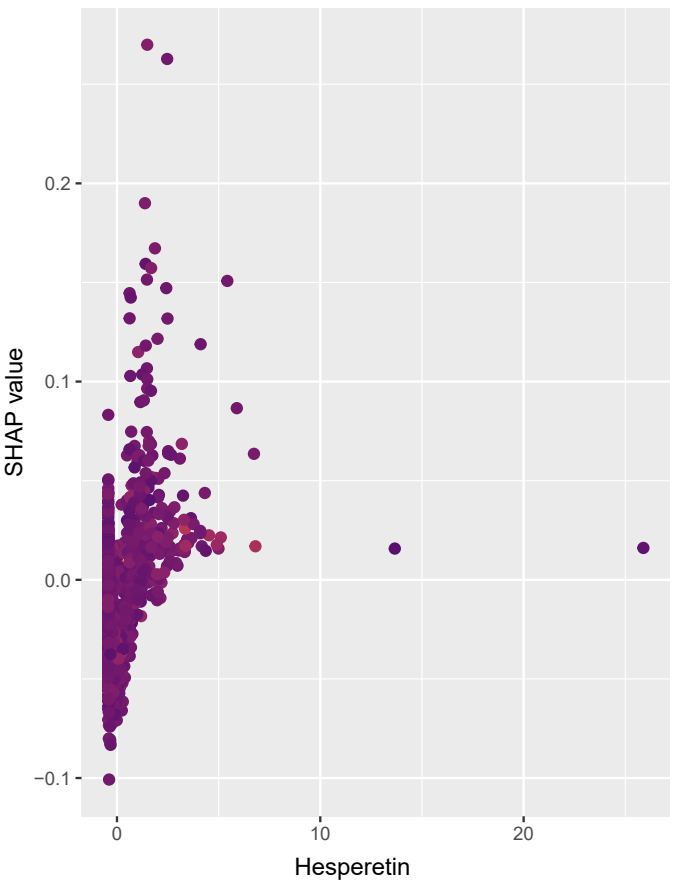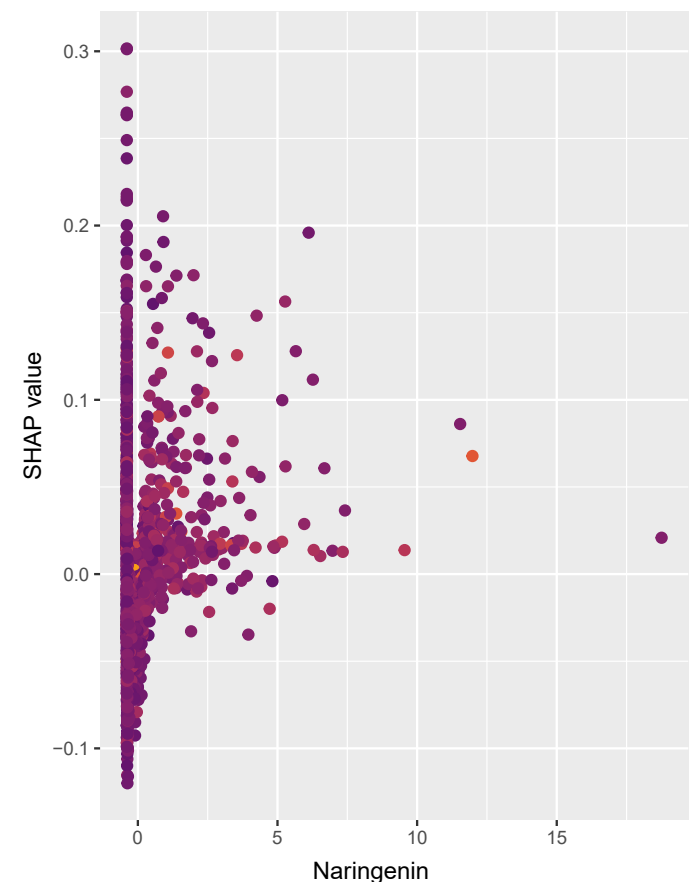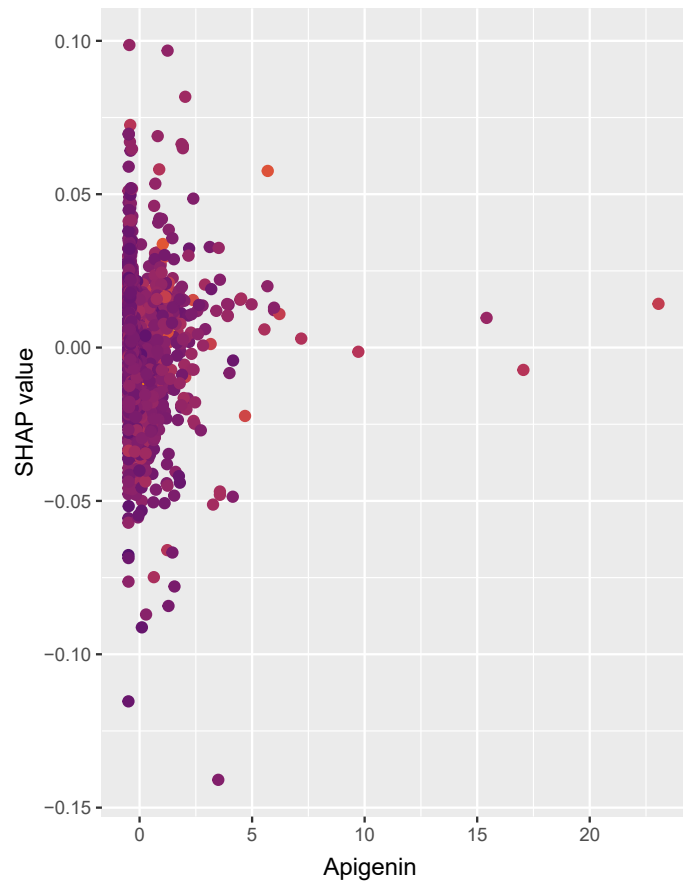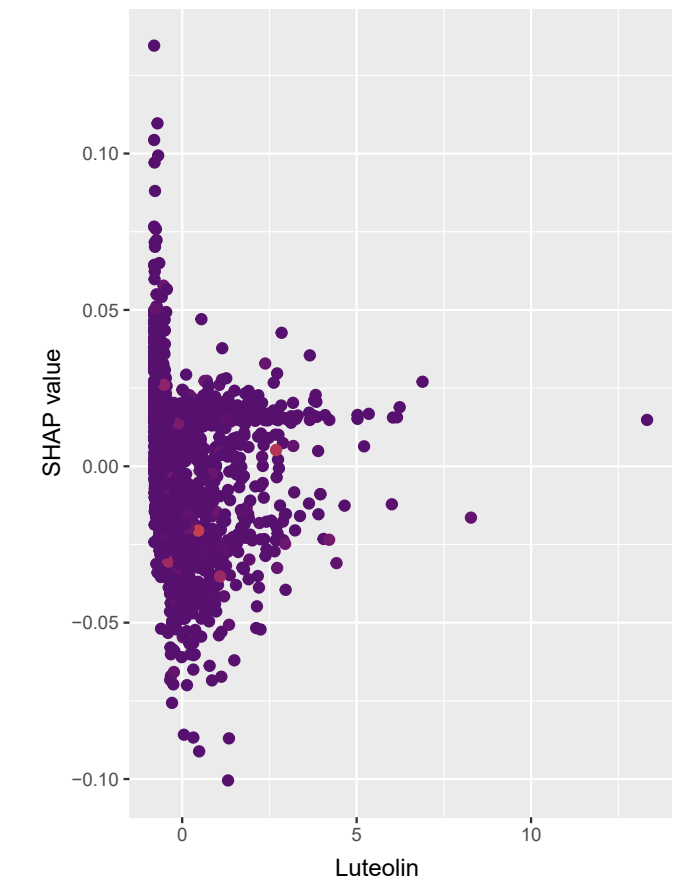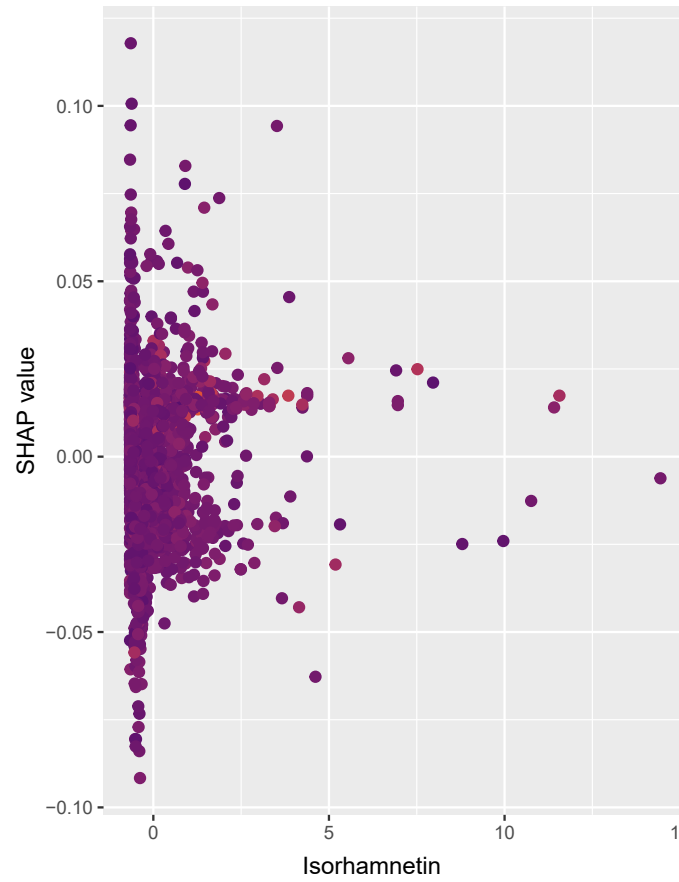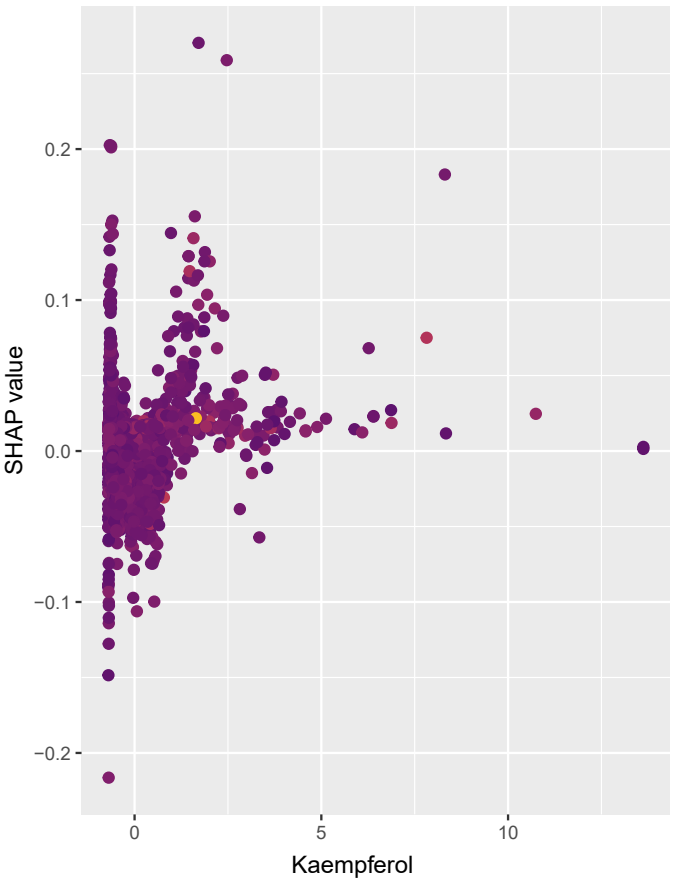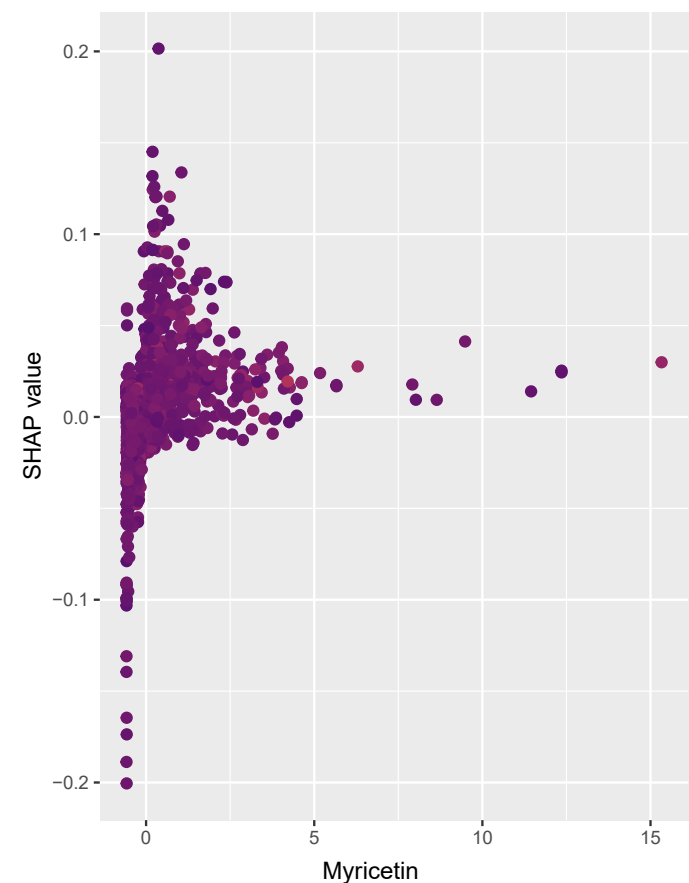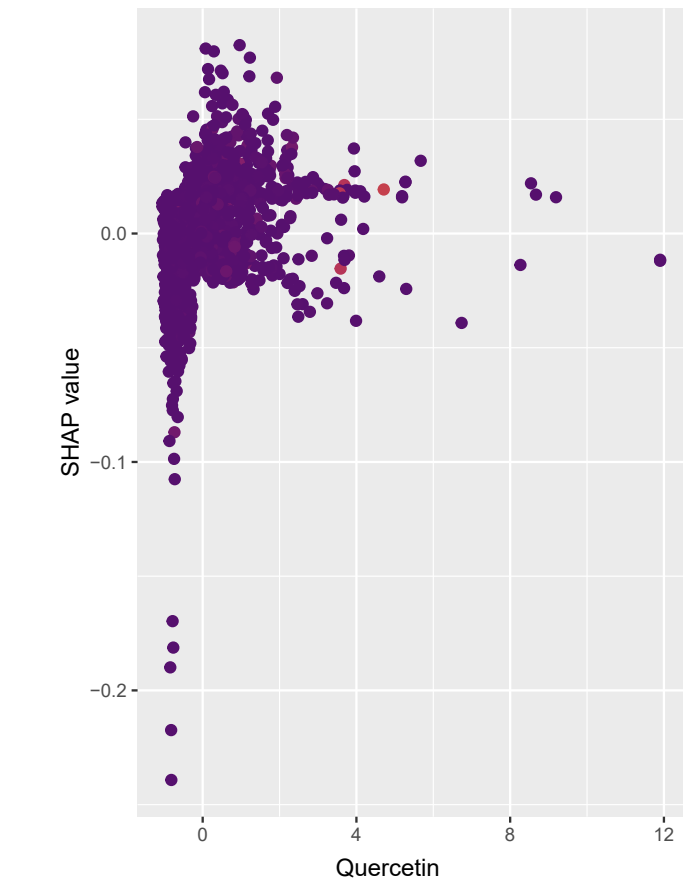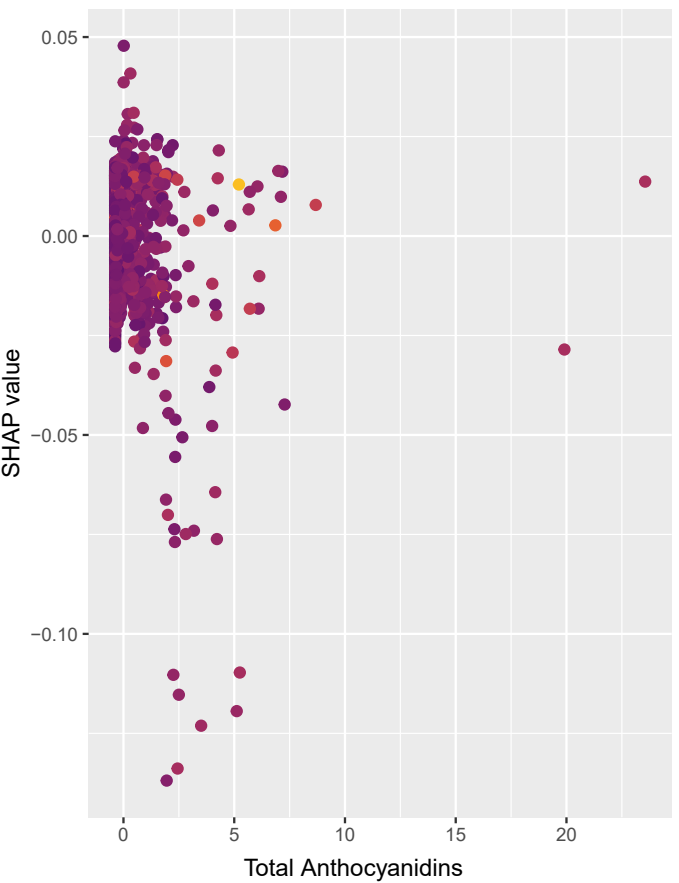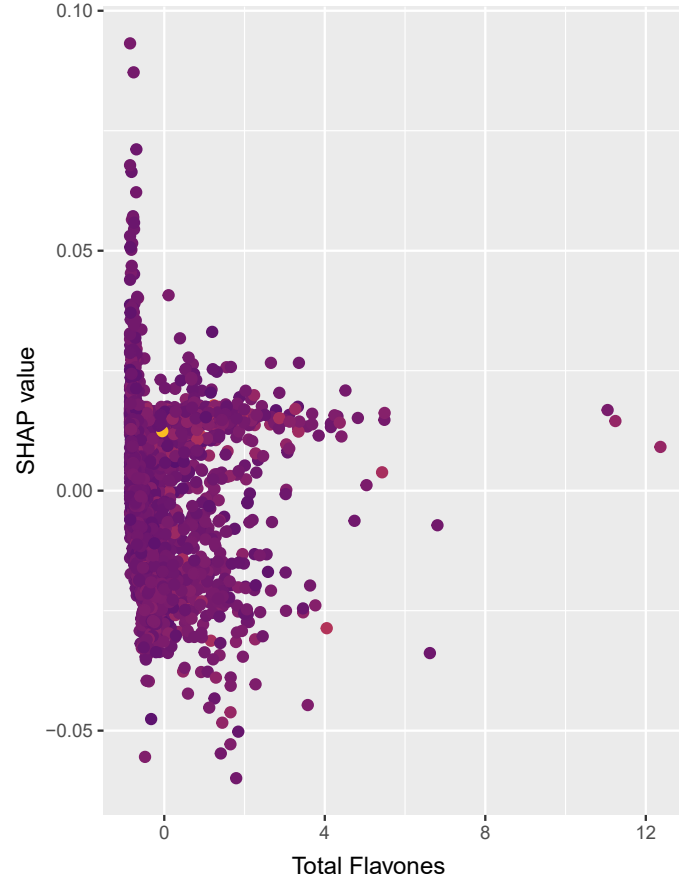

Supplement: Multimedia component 11 [file mmc11.pdf]
